# Prediction of LncRNA Subcellular Localization with Deep Learning from Sequence Features

**DOI:** 10.1038/s41598-018-34708-w

**Published:** 2018-11-06

**Authors:** Brian L. Gudenas, Liangjiang Wang

**Affiliations:** Department of Genetics and Biochemistry, Clemson University, Clemson, SC USA

## Abstract

Long non-coding RNAs are involved in biological processes throughout the cell including the nucleus, chromatin and cytosol. However, most lncRNAs remain unannotated and functional annotation of lncRNAs is difficult due to their low conservation and their tissue and developmentally specific expression. LncRNA subcellular localization is highly informative regarding its biological function, although it is difficult to discover because few prediction methods currently exist. While protein subcellular localization prediction is a well-established research field, lncRNA localization prediction is a novel research problem. We developed DeepLncRNA, a deep learning algorithm which predicts lncRNA subcellular localization directly from lncRNA transcript sequences. We analyzed 93 strand-specific RNA-seq samples of nuclear and cytosolic fractions from multiple cell types to identify differentially localized lncRNAs. We then extracted sequence-based features from the lncRNAs to construct our DeepLncRNA model, which achieved an accuracy of 72.4%, sensitivity of 83%, specificity of 62.4% and area under the receiver operating characteristic curve of 0.787. Our results suggest that primary sequence motifs are a major driving force in the subcellular localization of lncRNAs.

## Introduction

The inner workings of the cell are orchestrated by complex interactions between the products of DNA, both non-coding RNAs and proteins. This idea has superseded the view that proteins and their corresponding messenger RNAs (mRNAs) are solely responsible for cellular function. Non-coding RNAs are now known to be an integral functional system of the genome which are involved in crucial roles such as the regulation of gene expression. The most prevalent and one of the most functionally diverse classes of non-coding RNAs are the long non-coding RNAs (lncRNAs).

LncRNAs are large RNA transcripts which do not encode proteins and are estimated to outnumber protein-coding genes within the human genome^1^. However, lncRNAs are poorly conserved at the sequence level, which makes functional annotation difficult. LncRNAs perform a diverse repertoire of essential molecular functions, in many different subcellular locations^2^. However, determining the functional roles of lncRNAs experimentally is highly time-consuming and laborious. Like proteins, lncRNA functionality is dependent on proper subcellular localization. LncRNA transcripts can localize in many different places within the cell, including the chromatin, nucleus, cytoplasm and exosomes^3,4^. Knowing the localization patterns of lncRNAs allows the generalization of their biological functional. Therefore, the possibility to learn where a given lncRNA localizes would provide valuable information regarding its biological function as well as the RNA localization mechanism.

LncRNA subcellular localization is likely dependent on many factors, including sequence and structural motifs which can facilitate binding to proteins involved in localization^5^. Identification of structural motifs in lncRNAs is currently problematic both experimentally and computationally due to the high-level of complexity of intra-molecular organization that lncRNAs can exhibit^6^. However, sequence motifs in lncRNAs associated with subcellular localization have been identified such as the pentamer motif AGCCC which is highly associated with lncRNA nuclear localization^7^. Therefore, it is evident that motifs in the lncRNA primary sequence are involved in lncRNA subcellular localization. Obtaining lncRNA structural data is difficult, however, lncRNA transcript sequences are readily available.

Protein subcellular localization has been an active research area for decades and many localization motifs have been identified. These localization motifs either reside in the primary sequence, such as the N-terminal signal peptide associated with the secretory pathway, or within the 3D protein structure, such as DNA-binding domains in nuclear proteins. A well-known method for protein subcellular localization prediction is MultiLoc, a support vector machine (SVM) which uses sequence-derived features and achieved an average cross-species accuracy of 75%^8^. DeepLoc, a deep learning algorithm, recently achieved an accuracy of 91% on the same data set used by MultiLoc^9^. However, the proteins in this dataset have been found to be highly homologous and therefore might provide an overly-optimistic model evaluation. Using a more comprehensive dataset of proteins which localize to ten different subcellular locations, DeepLoc achieved an accuracy of 77%, while MultiLoc2, an upgraded version of MultiLoc, only achieved an accuracy of 55%^9^. Sequence-based features thus appear to be highly informative for protein subcellular localization and deep learning attains exceptional accuracy in comparison to other machine learning algorithms. Despite the well-established knowledge regarding protein localization prediction, we know relatively little about the prediction of lncRNA localization.

Our goal is to learn a model that predicts lncRNA subcellular localization directly from lncRNA nucleotide sequences. We have chosen to utilize a deep neural network (DNN), which have shown promise in many bioinformatics applications such as the annotation of non-coding variants and identification of enhancers^10,11^. Deep learning methods, such as DNNs, avoid the need to manually craft informative features and instead automatically learn high-level features through the iterative aggregation of features in each layer of the network. Since nuclear retention motifs have already been found in nuclear localized lncRNAs we expect differences in sequence composition between distinct nuclear and cytosolic lncRNAs^7^. Therefore, we used binary classification to learn how to discriminate between differentially localized nuclear and cytosolic lncRNAs. Our task is to predict the subcellular localization of lncRNAs based on their transcript sequence, therefore we named our algorithm DeepLncRNA, an acronym for “Deep Learning of Nuclear Classification of long non-coding RNAs”. We train our model on the sequences of differentially localized lncRNAs, which are either enriched in the nucleus or the cytosol. DeepLncRNA scans the lncRNA sequence, computing a range of k-mer frequencies and protein-binding motifs which are then used to predict the lncRNA localization.

Features were extracted from lncRNA transcript sequences for model construction; and therefore this methodology could be easily applied to any uncharacterized human lncRNAs. LncRNAs are lowly conserved between species, and a large fraction of human lncRNAs are primate specific^12,13^. Nevertheless, our model could be applicable to lncRNAs in closely related primates such as the chimpanzee or bonobo. This study represents one of the first steps in lncRNA subcellular localization prediction which will be a valuable resource for the functional annotation of this large, diverse and not yet fully understood class of non-coding genes.

## Methods

### Datasets

We analyzed paired-end strand-specific RNA-sequencing data from human cell lines from the ENCODE project^14^. Samples underwent cellular fractionation, to separate either the nucleus or cytosol, prior to RNA-seq. In total, we acquired 93 RNA-seq sampes from 14 human immortalized cell lines, of which 45 were from the cytosol and 48 from the nucleus. All cell lines were required to contain at least two samples from each cellular fraction. Samples underwent different RNA library protocols such as poly(A)+ (n = 62), total RNA (n = 8) or poly(A)- (n = 23). Using the total RNA and poly(A)- library protocols in addition to the standard poly(A)+ samples allows a complete transcriptomic analysis of lncRNAs, which are not all polyadenylated. All sample metadata as well as transcriptome alignment rates are displayed in Table S1.

Raw RNA-seq reads were mapped to the human transcriptome and quantified using Kallisto (v0.43.1)^15^. In total, ~6 billion reads were aligned to the human transcriptome (Ensembl v92, GrCh38)^16^. Differential transcript expression analysis between the nuclear and cytosolic fractions for each cell type was performed using Sleuth (R package, v0.29.0) which was shown to be superior to other methods at identifying differentially expressed transcripts^17^. If multiple RNA library protocols were used for a single cell type then we added this as a covariate when testing for differential transcript expression. LncRNAs were identified based on the gencode (v28) long non-coding RNA annotations for further analysis^18^. All Source code used in this work and the DeepLncRNA model are available at https://github.com/bgudenas/DeepLncRNA/.

### Identification of Differentially Localized Human LncRNAs

**Figure 1:**
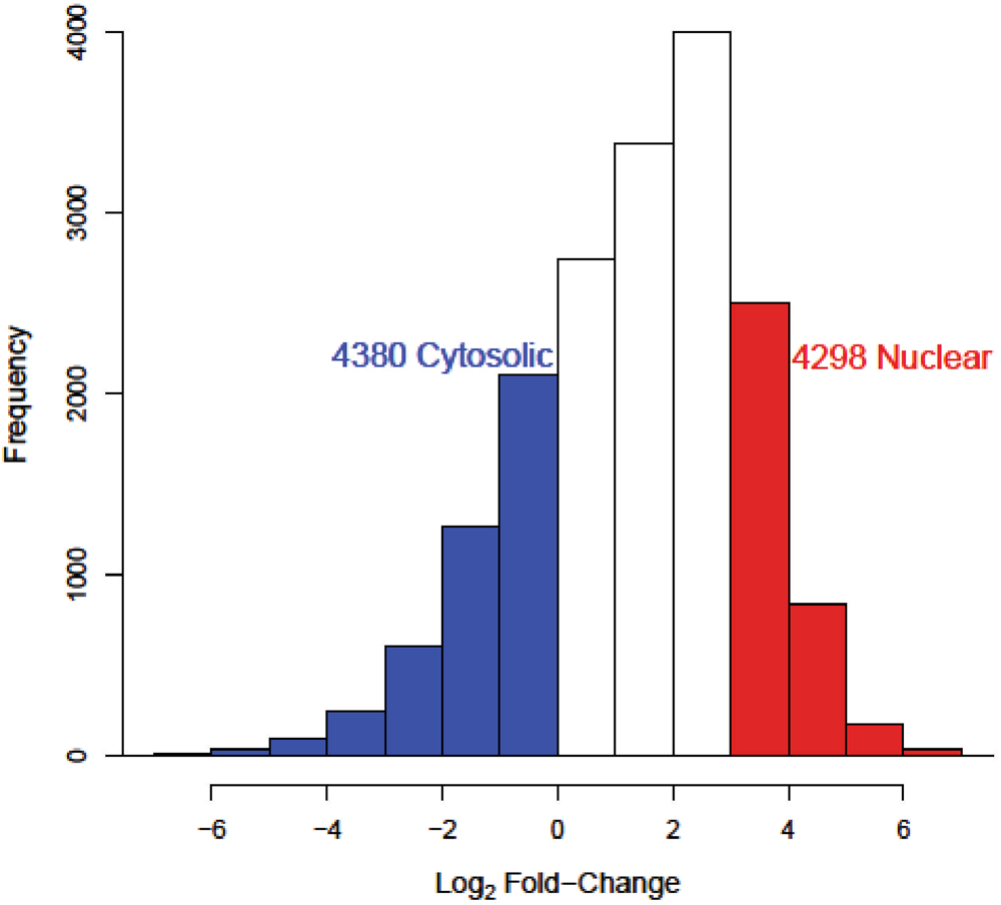
Distribution of the lncRNA nuclear to cytosolic transcript ratios. A histogram showing the log_2_ fold-change ratios for lncRNA transcripts (n = 18,068) detected across all cell types. Colored bars indicate differentially localized lncRNAs which passed fold-change thresholds (Cytosolic < 0; Nuclear > 2.8) resulting in a training set of 4380 cytosolic lncRNAs and 4298 nuclear lncRNAs.

We performed differential transcript expression to quantify the differences in lncRNA transcript abundances between the nuclear and cytosolic cellular fractions for each cell type. We aggregated the log2 fold-change values for each lncRNA across all cell types using a weighted average based on sample sizes per cell type. Computing the nuclear to cytosolic log_2_ fold-change allowed the examination of the distribution of lncRNA subcellular localization for over 18000 lncRNA transcripts (Fig. 1). In agreement with previous studies, we found lncRNAs to be predominantly enriched in the nucleus^19,20^. However, we do detect a large portion of lncRNAs (n = 4380) with transcript abundances higher in the cytosol than the nucleus (Fig. 1). Part of the nuclear skew of this distribution is likely explained by the fact that all lncRNAs, regardless of destination, must originate in the nucleus through the act of transcription. Furthermore, once transcribed the export of lncRNAs from the nucleus to the cytoplasm must take some amount of time due to the export mechanism, such as assembly of ribonucleoprotein complexes and recruitment of exporters^21^. Due to these two factors we expect the median lncRNA nuclear to cytosol transcript ratio to be greater than zero and indeed the median log_2_ fold-change was 1.6. Therefore, since our distribution is not centered at zero, like a standard differential expression test, we must adjust the commonly used symmetric log2 fold-change threshold to classify differential expression. To account for the nuclear skew of transcript ratios we selected new log_2_ fold-change thresholds, corresponding to the first and fourth quartile, to signify differential localization (cytosolic < 0, nuclear > 2.8). Applying these fold-change thresholds to our data resulted in a balanced dataset of 4380 cytosolic lncRNAs and 4298 nuclear lncRNAs. The dataset was then split into training, validation and testing sets using a randomized 70/15/15 percent split.

### Extraction of Sequence Features from LncRNAs

**Figure 2:**
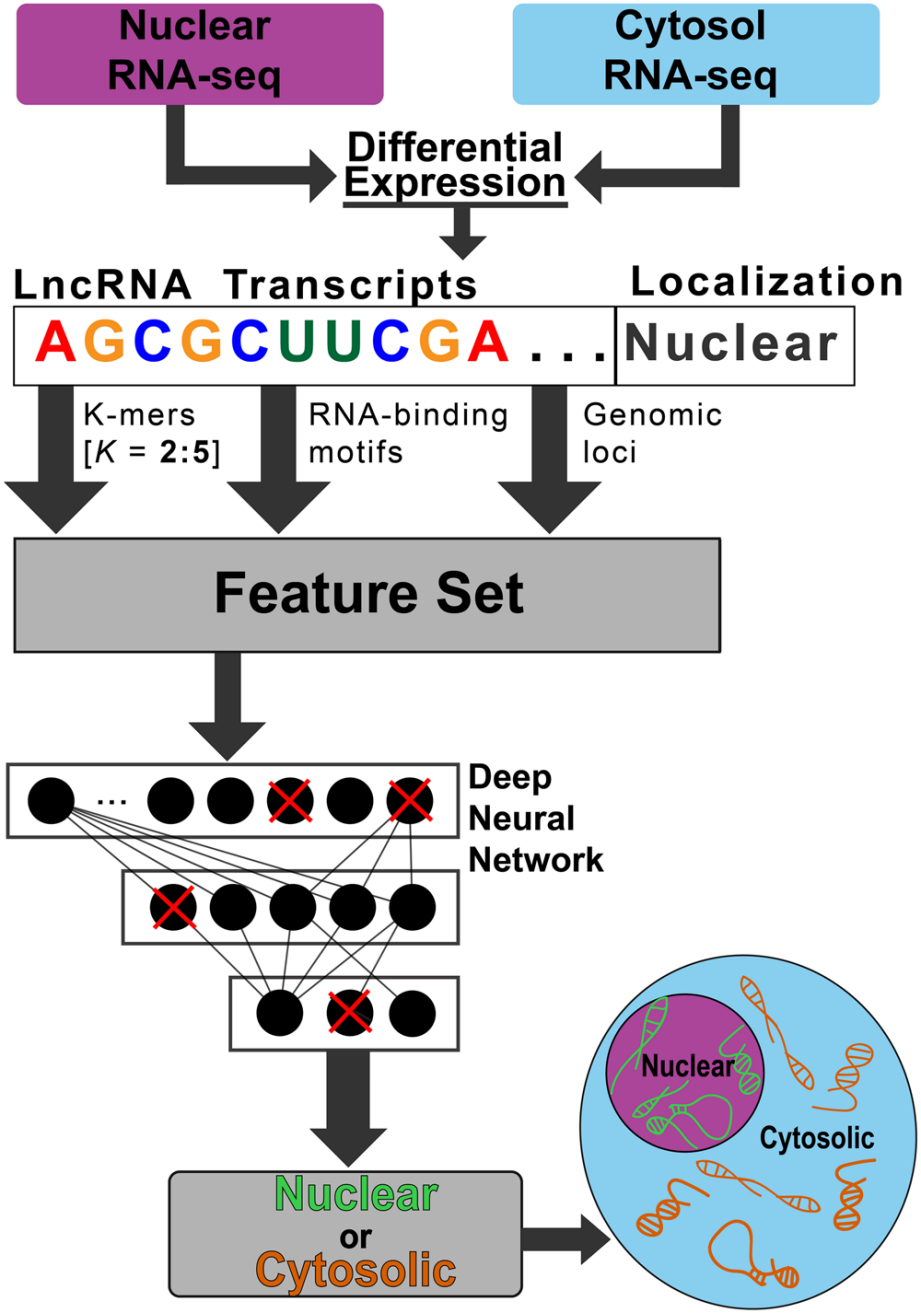
Overview of the DeepLncRNA algorithm.

To derive sequence-based features of uniform length from transcript sequences of variable length we counted k-mers. Using the lncRNA cDNA sequences of the differentially localized lncRNAs we computed a k-mer frequency matrix, containing the frequency of all possible oligonucleotides for *k* equal to two through five resulting in (4^2^+4^3^+4^4^+4^5^) 1360 k-mer features. In addition, the genomic loci of lncRNAs are known to be important regarding their functionality which is why lncRNAs are classified based on their genomic context such as intergenic, antisense or sense lncRNAs^22^. Therefore, we added additional features representing these major lncRNA subtypes based on the transcript annotations from ENSEMBL. We also added the chromosome the lncRNA is located on to further capture any effects of its genomic location. Lastly, the binding of RNA by proteins represents a possible mechanism in which lncRNAs may be localized. Therefore, we added features representing the presence of known RNA-binding protein motifs which were obtained from the CISBP—RNA database^23^. Matches were counted using a sliding-window approach, and a match was scored if the sub-sequence obtained a log-likelihood position weight matrix (PWM) score greater than 80% of the maximal PWM score^24^. In total, we obtained 1582 sequence-based features which are the inputs for DeepLncRNA (Fig. 2). The DeepLncRNA dataset is provided in Table S2.

### Deep Neural Network Model

DeepLncRNA is a feed-forward multi-layer deep neural network. The architecture consists of one input layer, three hidden layers using the rectified linear unit activation function and a softmax output layer. Hidden layer dropout was used to randomly mask half of the connections in each layer during training of the DNN which reduces the propensity for overfitting. Input dropout was also applied which randomly masks some of the hidden units in each layer to increase the generalizability of the model. Furthermore, regularization was applied using the L1 and L2 weight penalties to the cost function. All model parameter values were selected using a random search over all possible parameter combinations seeking to minimize the misclassification rate on the validation set. DeepLncRNA was trained with stochastic gradient descent using the backpropagation algorithm which adjusts network weights by minimizing the error between the response variable and the predicted output. DeepLncRNA was built using the h2o R package^25^.

### Evaluation Criteria

In this work, we develop a DeepLncRNA to identify lncRNAs to be enriched in the nucleus (positive class) or cytosol (negative class). We use the common machine learning metrics such as accuracy, sensitivity, specificity and Matthews correlation coefficient for classifier performance evaluation. TP is the number of true positives; TN is the number of true negatives; FP is the number of false positives; and FN is the number of false negatives.

1 $$Accuracy=\,\frac{TP+TN}{TP+TN+FP+FN}$$

2 $${Sensitivity}=\frac{TP}{TP+FN}$$

3 $${Specificity}=\frac{TN}{TN+FP}$$

4 $$MCC=\frac{TP\times TN-FP\times FN}{\sqrt{(TP+FP)(TP+FN)(TN+FP)(TN+FN)}}$$

5 $$F1=2\times \,\frac{\frac{TP}{TP+FP}\,\times \frac{TP}{FN+TP}}{\frac{TP}{TP+FP}+\frac{TP}{FN+TP}}$$

## Results

**Figure 3:**
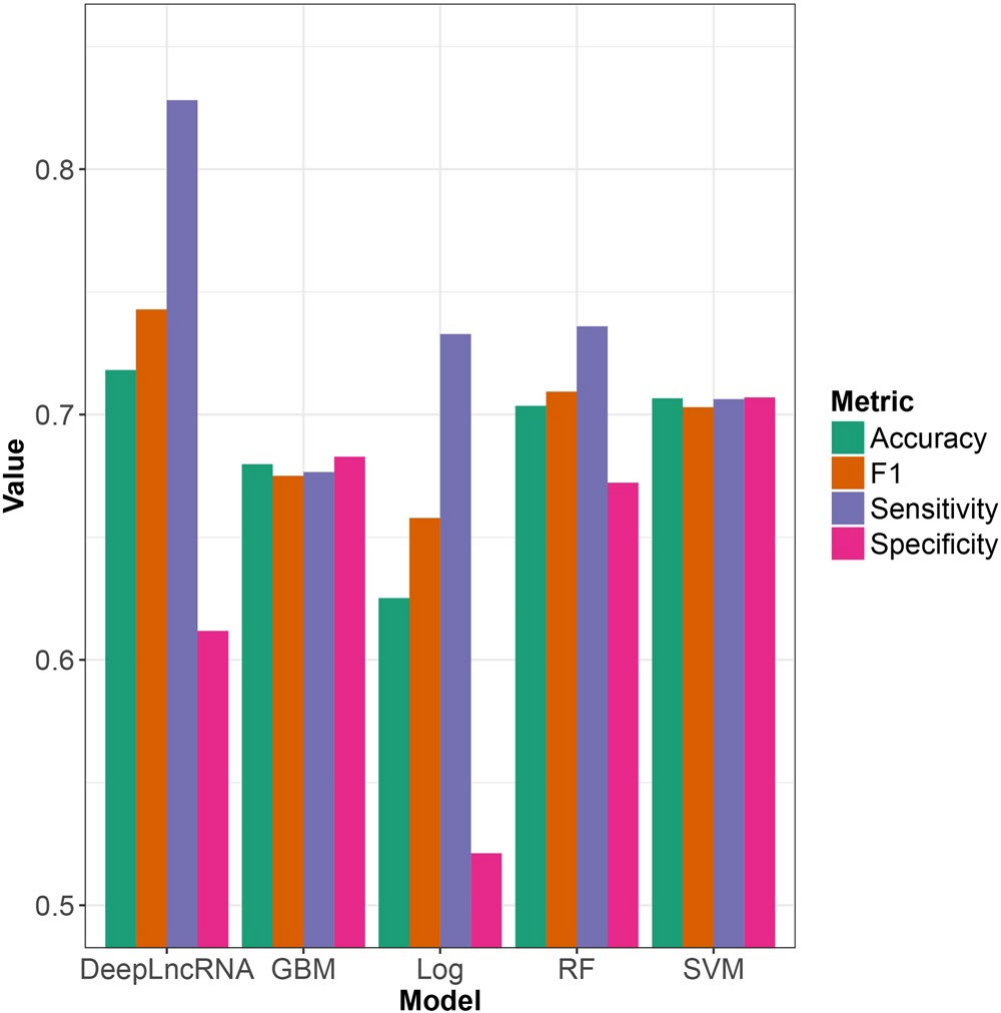
Model selection based on performance metrics on the validation set. The performance metrics of DeepLncRNA, stochastic gradient boosting (GBM), boosted logistic regression (Log), random forest (RF), support vector machine (SVM) on the validation set.

To evaluate the performance of DeepLncRNA we compared it to other advanced machine learning algorithms. We compared DeepLncRNA with four other machine learning algorithms (Fig. 3). Based on all measures, except specificity, DeepLncRNA achieved superior performance. The ability to abstract complex non-linear features does appear to enhance the performance of DeepLncRNA compared to the other machine learning algorithms.

**Figure 4:**
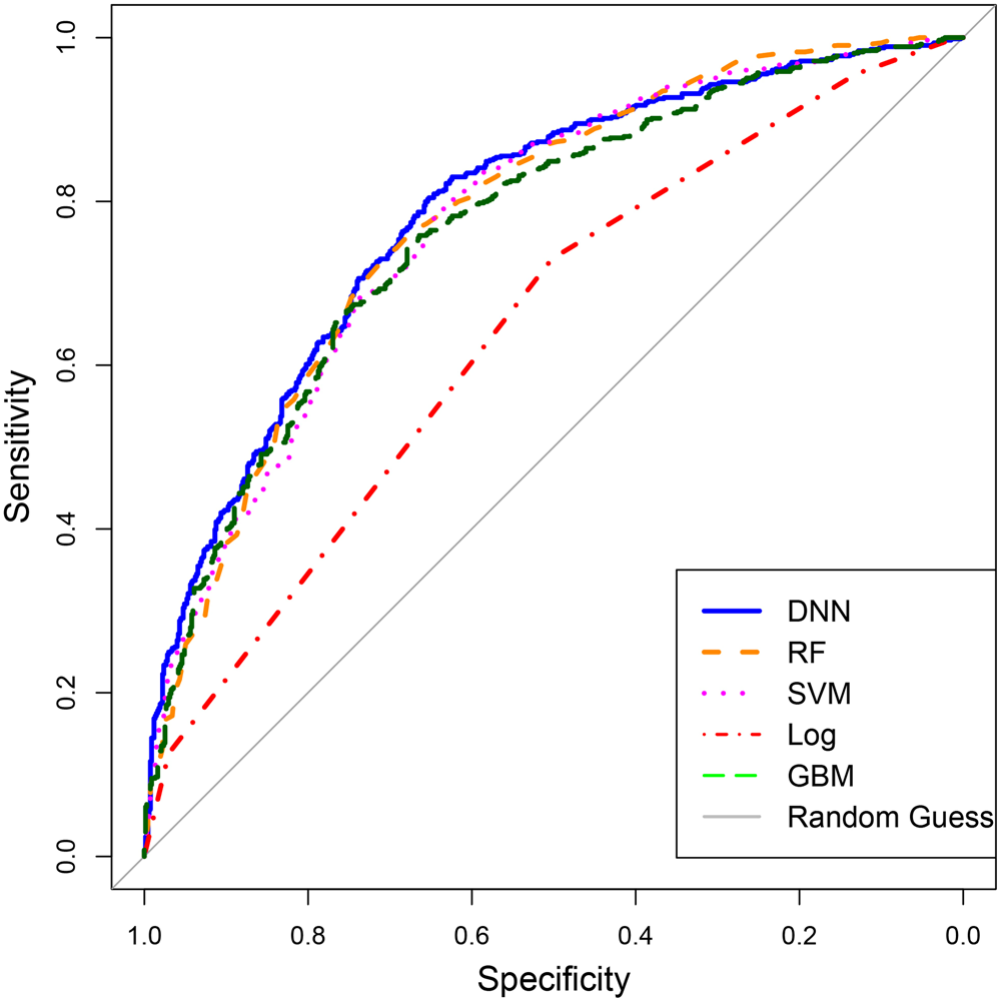
ROC curve performance comparison on the test set.

Model parameters were selected based on the maximization of accuracy on the validation set. Since DeepLncRNA has more parameters than the other models it is possibly an over-optimistic evaluation of its accuracy. Therefore, we generated ROC curves on the unseen test set for all these models (Fig. 4). The ROC curve shows DeepLncRNA has the highest discriminatory power between the nuclear and cytosolic lncRNAs. Furthermore, we compared DeepLncRNA to the other machine learning models using a range of performance metrics and found DeepLncRNA achieved superior performance on every metric except specificity (Table 1). While DeepLncRNA obtained a specificity lower than that of other models, its sensitivity is 10% higher than the next model, boosted logistic regression. Based on the more comprehensive metrics such as accuracy, F1, AUC and MCC shown here we conclude that DeepLncRNA is the best model for the prediction of lncRNA subcellular localization.

**Table 1:** Performance metrics on the test set.

Model	Accuracy	Sensitivity	Specificity	F1	AUC	MCC
GBM	0.703	0.693	**0**.**712**	0.693	0.766	0.405
Log	0.625	0.733	0.521	0.658	0.643	0.238
RF	0.717	0.765	0.672	0.723	0.779	0.437
SVM	0.699	0.719	0.681	0.698	0.774	0.399
DNN	**0**.**724**	**0**.**83**	0.624	**0**.**744**	**0**.**787**	**0**.**451**

The features utilized for model construction consist of three major sets, which are sequence k-mers, known RNA-binding protein motif sites and genomic characteristics. To assess the importance of the three different feature sets we calculated their total relative feature importance. As a percentage of the total feature importance, the k-mer, RNA-binding protein motif and genomic features provide 90%, 8.6% and 1.4%, respectively, of the total feature importance (Table S3). However, 86% of the features are k-mers, therefore, by normalizing the total relative feature importance of each group by the number of features of each set, the k-mer, RNA-binding motif and genomic features have 0.30, 0.27 and 0.34, respectively, normalized relative feature importance (Table S3). These results indicate that the genomic features on average provide more information per feature than the k-mers. In fact, the most informative feature of the whole dataset, based on relative feature importance, is whether the lncRNA is located sense to a proximal protein-coding gene (Table S3). Furthermore, these results show that the inclusion of non-sequence based features, such as genomic characteristics, are beneficial for the prediction of lncRNA subcellular localization.

**Figure 5:**
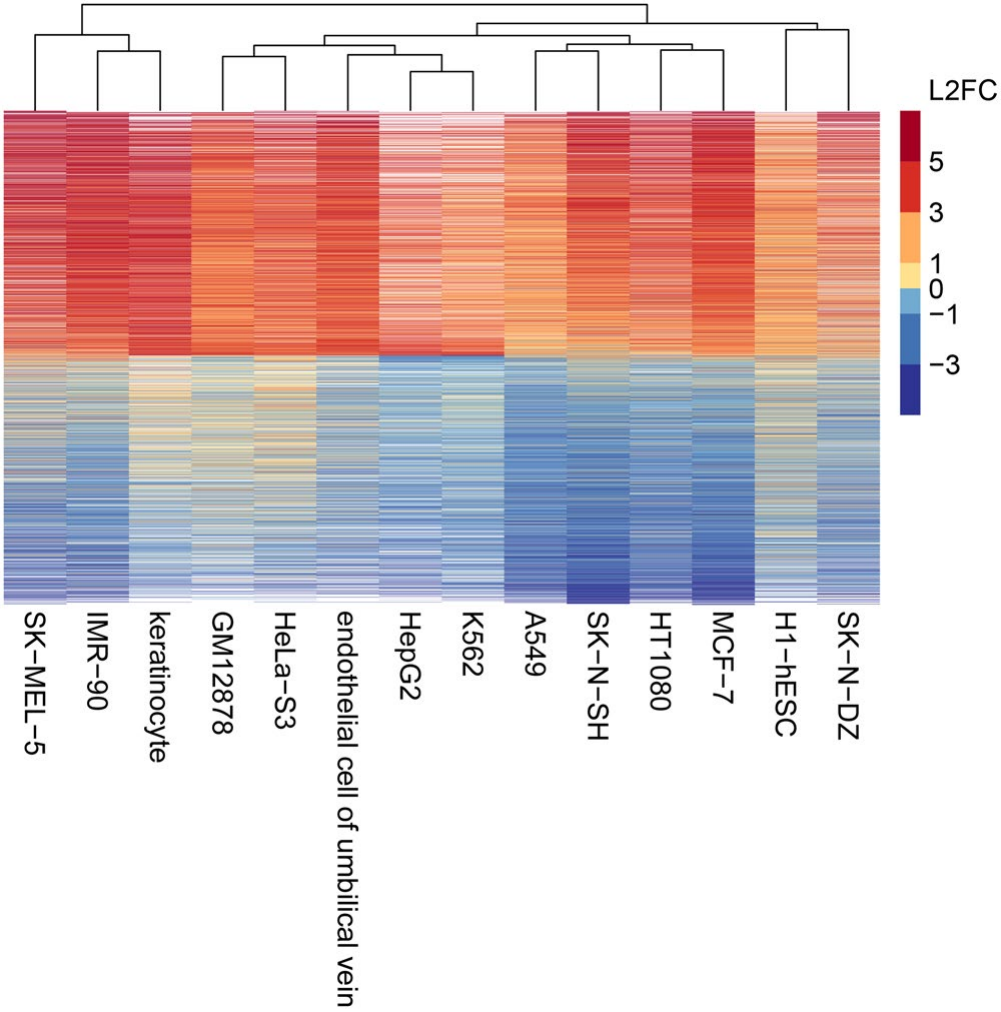
Heatmap of lncRNA nuclear to cytosolic transcript ratios across cell types. Each bar is a lncRNA transcript colored according to its nuclear to cytosolic log_2_ fold-change (L2FC) in the respective cell type, white bars indicate the lncRNA was not detected in that cell type. Cell types were then clustered based on their lncRNA localization patterns.

To show that DeepLncRNA can be applied to lncRNAs in cell types other than the ones used here for training, we examined the role that cell type has on lncRNA subcellular localization. Different cell types have distinct gene expression profiles which could affect the abundance of the export machinery, such as exporter proteins, needed for specific lncRNAs to exit the nucleus. Therefore, we visualized the conservation of lncRNA subcellular localization across all cell types used in this study (Fig. 5). Despite the vast differences in tissue types, lncRNA subcellular localization appears highly conserved across cell type. Since the subcellular localization of a lncRNA is not dependent on cell type, our model is applicable to all human lncRNAs. However, for a small number of lncRNAs there are changes in subcellular localization between certain cell types. This suggests it may be beneficial to add cell type specific features in the future for the prediction of lncRNA subcellular localization.

**Figure 6:**
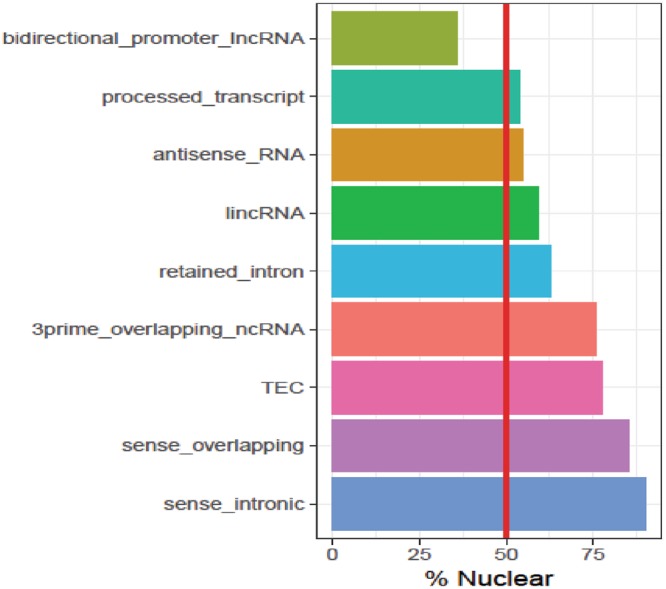
Percent of annotated lncRNAs predicted to localize in the nucleus. DeepLncRNA predictions of the localization of all annotated lncRNAs grouped by lncRNA biotype. Each bar represents the total percent of lncRNAs in that biotype that are predicted to be localized in the nucleus. The red vertical line represents the boundary between a predominantly cytosolic enriched or nuclear enriched biotype.

To examine the subcellular localization properties of different subcategories of lncRNAs we used DeepLncRNA to predict the subcellular localization of all annotated human lncRNAs, excluding any lncRNAs in our training set. In total, we predicted the localization of over 20,000 lncRNAs which we then grouped by gene biotype and evaluated based on the proportion which localize to the nucleus (Fig. 6). Intriguingly, we observed drastically different proportions of nuclear localization between lncRNA biotypes. Most notably, sense intronic lncRNAs, which reside in the intron of a protein-coding gene, are almost entirely predicted to be enriched in the nucleus. In fact, sense overlapping lncRNAs which can share exons with protein-coding genes are also predicted to be highly nuclear. Thus, both types of sense lncRNAs appear to be highly nuclear which may suggest they predominantly function in the cis-regulation of their embedded protein-coding gene. Almost half of antisense lncRNAs are predicted to be enriched in the cytosol. This is compatible with the fact that many antisense lncRNAs are known to increase the stability of their cognate mRNA by protection from miRNA in the cytoplasm^26^. Next, we compared the predictions of DeepLncRNA with experimental results from RNA profiling studies of lncRNA subcellular localization.

**Figure 7:**
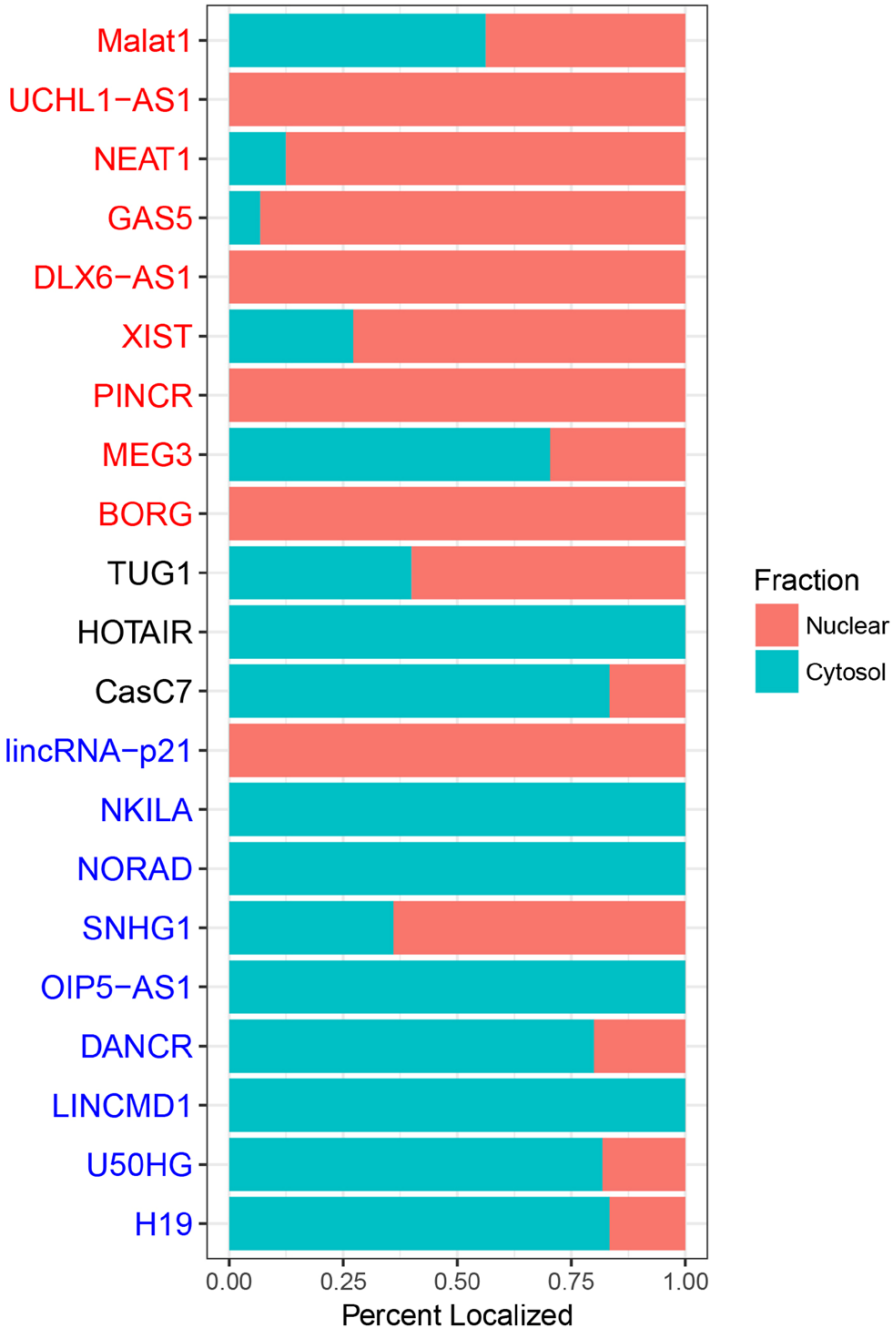
DeepLncRNA predictions on lncRNAs with known subcellular localizations. A stacked bar plot showing the percent of lncRNA transcripts predicted to localize to a specific subcellular fraction. LncRNA gene names colored by (red, black and blue) represent nuclear, dual-localized and cytoplasmic lncRNAs, respectively, identified in experimental studies (Table S4).

Several lncRNAs have already had their subcellular localization studied through experimental approaches such as fluorescent *in situ* hybridization of RNA^27^. From the current literature we curated a list of twenty-one lncRNAs with known subcellular localizations, including three lncRNAs which were found to be dual-localized in both subcellular fractions (Table S4). However, many of these differentially localized lncRNAs were present in our dataset, therefore, we removed all of them from the training and validation set and recreated DeepLncRNA using the exact same parameters originally used. We then used the new version of DeepLncRNA to predict the subcellular localization of these lncRNAs which have had their localization experimentally tested yet have never been seen by our model (Fig. 7). DeepLncRNA correctly predicted 7 out of 9 nuclear lncRNAs and 7 out 9 cytoplasmic lncRNAs, based on greater than 50% probability for their respective fraction. Despite being not trained on dual-localized lncRNAs, DeepLncRNA correctly predicted that three such lncRNAs (TUG1, HOTAIR, and CasC7) are present in the cytoplasm. The nuclear lncRNA BORG is a mouse lncRNA, and DeepLncRNA correctly predicted the nuclear retention of BORG (Fig. 7). The results suggest that DeepLncRNA learned generalizability from the sequence-based features, and can predict the lncRNA subcellular localization of new lncRNAs.

**Figure 8:**
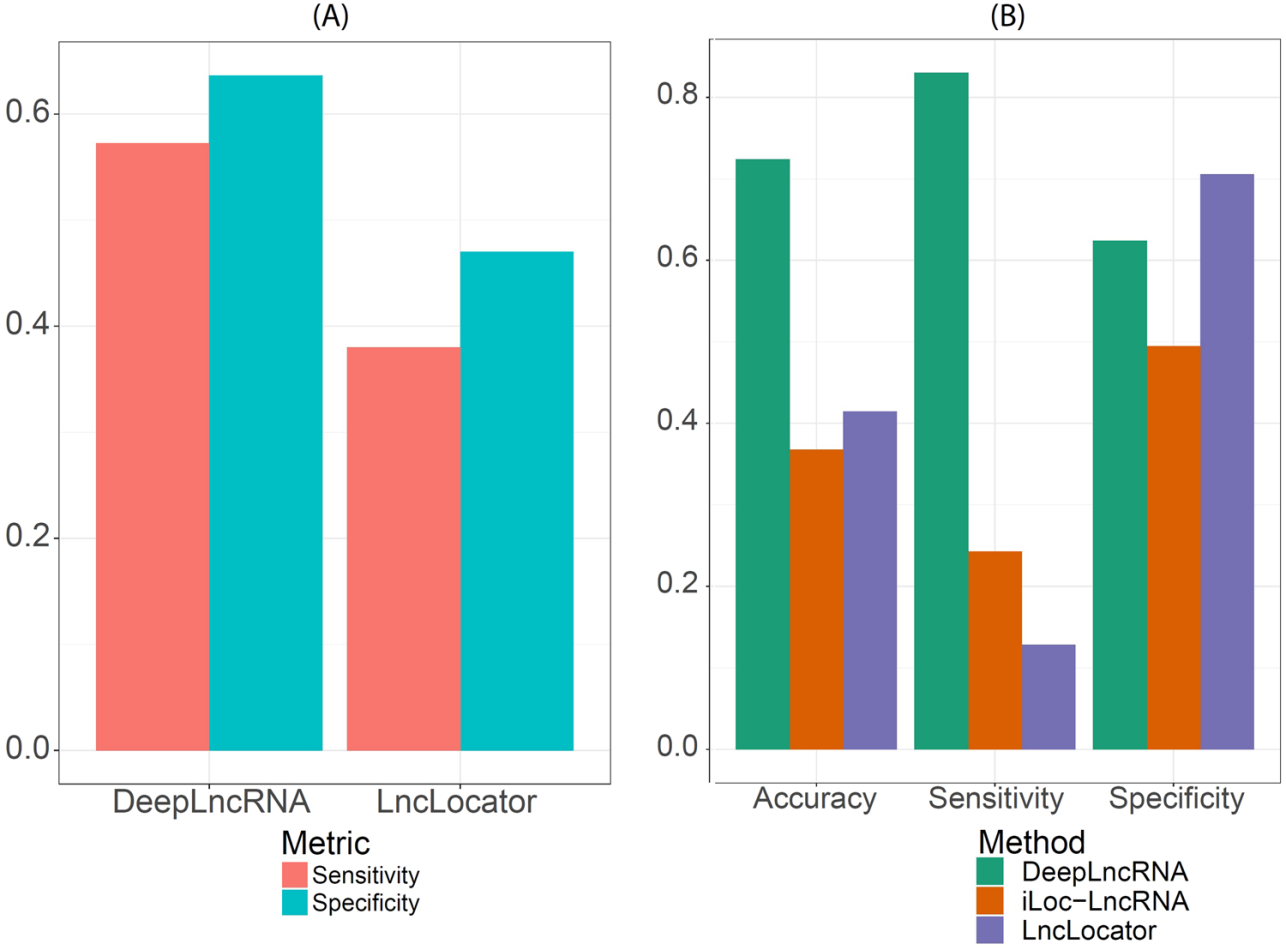
Method comparison. (**A**) Evaluation of the ability to predict nuclear lncRNAs (sensitivity) and cytosolic lncRNAs (specificity) achieved by LncLocator and DeepLncRNA on the LncLocator test set. (**B**) Performance comparison of LncLocator, iLoc-LncRNA and DeepLncRNA on the DeepLncRNA test set.

To evaluate the performance of DeepLncRNA on an independent unseen test set, we compared it to another lncRNA subcellular localization method, LncLocator^28^. LncLocator is a sequence-based method which uses a stacked autoencoder to derive high level features for an ensemble of machine learning models to predict five subcellular localizations. Therefore, we compared the performance of LncLocator and DeepLncRNA on the two subcellular localizations which both methods predict, nuclear and cytosolic localizations. Using DeepLncRNA we predicted the localization of the 152 nuclear and 91 cytosolic lncRNAs used in the LncLocator test set (Fig. 8A). Compared to LncLocator, DeepLncRNA achieves superior performance in the ability to predict nuclear and cytosolic lncRNAs (Fig. 8A). Interestingly, approximately half of the lncRNAs in this dataset are mouse lncRNAs indicating that DeepLncRNA, which was trained only on human lncRNAs, has learned generalizable features for lncRNA subcellular localization.

In addition, we also compared DeepLncRNA to another recently published model, named iLoc-LncRNA, which utilizes sequence octamers to derive pseudo K-tuple nucleotide compositions as features for a multi-class SVM model^29^. However, both iLoc-LncRNA and Lnclocator were built using less than one thousand lncRNAs from the RNALocate database, which is relatively small compared to our dataset of over eight thousand lncRNAs^30^. Therefore, we evaluated both iLoc-LncRNA and LncLocator on the test set used to evaluate DeepLncRNA (Fig. 8B). DeepLncRNA obtains superior accuracy and sensitivity, which is the capacity to correctly classify nuclear lncRNAs, relative to the other models. Lnclocator attains the highest specificity but at the cost of a low sensitivity. It is important to note that both of these other models are multi-class predictors, which predict additional subcellular localizations such as the ribosome and exosomes, unlike DeepLncRNA, which currently only predicts nuclear and cytosolic localization. However, based on the number of lncRNAs in the RNALocate database as well as single-cell imaging studies, the nucleus and cytosol appear to be the predominant destinations of lncRNA subcellular localization^27,30^.

## Conclusion

In conclusion, we developed DeepLncRNA, a deep learning algorithm which predicts lncRNA subcellular localization directly from lncRNA transcript sequences. DeepLncRNA obtained superior accuracy relative to other state-of-the-art machine learning algorithms and represents a major advancement in lncRNA subcellular localization prediction. The high accuracy of DeepLncRNA indicates that lncRNA primary sequence motifs play a large role in subcellular localization. We predicted the subcellular localization of all annotated human lncRNAs, finding different biotypes possess distinct subcellular localization properties. DeepLncRNA also correctly predicted the localization of more than 75% of a manually curated list of lncRNAs with experimentally validated localizations. In addition, DeepLncRNA was superior in the prediction of nuclear and cytosolic lncRNAs when compared to other recent methods. In the future, lncRNA subcellular localization prediction will enable the examination of the role disease-associated point mutations and copy-number variants have on lncRNA function. Since the number of lncRNAs is expanding we expect DeepLncRNA to play a pivotal role in the functional annotation of lncRNAs. User-friendly and publicly accessible web-servers represent the future of useful and accessible models and we will make efforts in our future work to provide a web-server for the methodology presented in this paper.

## Electronic supplementary material

Supplemental Tables

Dataset 1

## Data Availability

All data generated or analyzed during this study are included in this published article and its Supplementary Information files. All Source code used in this work and the DeepLncRNA model are available at https://github.com/bgudenas/DeepLncRNA/.
