# Supplementary material for: Prediction of LncRNA Subcellular Localization with Deep Learning from Sequence Features
Source: Sci Rep. 2018 Nov 6;8:16385. doi: 10.1038/s41598-018-34708-w (PMC6219567; doi:10.1038/s41598-018-34708-w)
Supplement: Supplementary file 1 — Supplemental Tables [file 41598_2018_34708_MOESM1_ESM.pdf]

Supplemental Tables

Prediction of LncRNA Subcellular Localization with Deep Learning from Sequence Features

Brian L. Gudenas and Liangjiang Wang*

Department of Genetics and Biochemistry, Clemson University, Clemson, SC, 29634, USA

* Corresponding Author

Email: liangjw@clemson.edu

RNA-Seq Sample Information

	ENCODE Sample ID	Cell Fraction	Cell Line	Sequencing Platform	Read Length	Total Reads	Reads Aligned
1	ENCFF000HCB	cytosol	IMR-90	HiSeq 2000	101	114006059	107141388
2	ENCFF000HCI	cytosol	IMR-90	HiSeq 2000	101	144283810	135925697
3	ENCFF000ICF	cytosol	keratinocyte	Illumina Genome Analyzer IIx	76	65330831	11643795
4	ENCFF447UEE	cytosol	HT1080	HiSeq 2000	101	96607714	90073609
5	ENCFF331FNA	cytosol	HT1080	HiSeq 2000	101	97861756	91040843
6	ENCFF000ELQ	nucleus	A549	HiSeq 2000	101	101864205	76751734
7	ENCFF000EMA	nucleus	A549	HiSeq 2000	101	184501699	152227603
8	ENCFF000IDC	cytosol	keratinocyte	Illumina Genome Analyzer IIx	76	98244162	84822309
9	ENCFF000IDF	cytosol	keratinocyte	Illumina Genome Analyzer IIx	76	108353593	97525872
10	ENCFF000FSU	nucleus	HeLa-S3	Illumina Genome Analyzer IIx	76	76400529	53722548
11	ENCFF000FTB	nucleus	HeLa-S3	Illumina Genome Analyzer IIx	76	105221091	70361733
12	ENCFF000IOD	nucleus	SK-N-SH	HiSeq 2000	101	149659336	116691980
13	ENCFF000IOP	nucleus	SK-N-SH	HiSeq 2000	101	243453503	180480071
14	ENCFF000EKW	cytosol	A549	HiSeq 2000	101	138533166	127720956
15	ENCFF000ELC	cytosol	A549	HiSeq 2000	101	154025915	141547131
16	ENCFF000HDD	nucleus	IMR-90	HiSeq 2000	101	167746727	137928851
17	ENCFF000HDE	nucleus	IMR-90	HiSeq 2000	101	171005868	125640076
18	ENCFF000EZF	cytosol	GM12878	Illumina Genome Analyzer IIx	76	12732173	10929927
19	ENCFF000EZH	cytosol	GM12878	Illumina Genome Analyzer IIx	76	127696795	108008678
20	ENCFF000EXH	cytosol	GM12878	Illumina Genome Analyzer IIx	76	103469359	13032346
21	ENCFF000EXI	cytosol	GM12878	Illumina Genome Analyzer IIx	76	105554104	15423816
22	ENCFF000GAF	nucleus	HepG2	Illumina Genome Analyzer IIx	76	102421616	67124080
23	ENCFF000GAH	nucleus	HepG2	Illumina Genome Analyzer IIx	76	86000652	56050222
24	ENCFF000FRY	nucleus	HeLa-S3	Illumina Genome Analyzer IIx	76	85363426	27177874
25	ENCFF000FSA	nucleus	HeLa-S3	Illumina Genome Analyzer IIx	76	104753832	28636006
26	ENCFF000FXU	cytosol	HepG2	Illumina Genome Analyzer IIx	76	112502244	103796419
27	ENCFF000FYG	cytosol	HepG2	Illumina Genome Analyzer IIx	76	111866942	102721580
28	ENCFF000FQK	cytosol	HeLa-S3	Illumina Genome Analyzer IIx	76	121136671	106966517
29	ENCFF000FQW	cytosol	HeLa-S3	Illumina Genome Analyzer IIx	76	104489603	94358101
30	ENCFF901DUO	cytosol	HepG2	HiSeq 2000	50	31719232	30247925
31	ENCFF792JEE	cytosol	HepG2	HiSeq 2000	50	24755754	23625699
32	ENCFF966SGR	cytosol	K562	HiSeq 2000	50	49792986	42678064
33	ENCFF376BGG	cytosol	K562	HiSeq 2000	50	43658969	38312110
34	ENCFF000FCG	nucleus	GM12878	Illumina Genome Analyzer IIx	76	128402941	76955641
35	ENCFF000FCH	nucleus	GM12878	Illumina Genome Analyzer IIx	76	116517502	65595488
36	ENCFF000FGL	cytosol	H1-hESC	Illumina Genome Analyzer IIx	76	60520880	9647729
37	ENCFF252KFX	nucleus	HepG2	HiSeq 2000	50	19884506	15631625
38	ENCFF710WZN	nucleus	HepG2	HiSeq 2000	50	21059227	16093349
39	ENCFF268FHH	cytosol	HepG2	HiSeq 2000	50	30564649	26924290
40	ENCFF196RCZ	cytosol	HepG2	HiSeq 2000	50	32552634	28298715
41	ENCFF000HOC	nucleus	K562	Illumina Genome Analyzer IIx	76	117113622	74578479
42	ENCFF000HOD	nucleus	K562	Illumina Genome Analyzer IIx	76	105769104	65878880
43	ENCFF470ZTS	cytosol	SK-MEL-5	HiSeq 2000	101	95167015	89049195
44	ENCFF687XKK	cytosol	SK-MEL-5	HiSeq 2000	101	86793652	81442068
45	ENCFF993CAL	nucleus	SK-N-DZ	HiSeq 2000	101	88098315	54736849
46	ENCFF482SFO	nucleus	SK-N-DZ	HiSeq 2000	101	78355317	42515227
47	ENCFF394ODW	nucleus	SK-MEL-5	HiSeq 2000	101	77069916	51602776
48	ENCFF005NLJ	nucleus	SK-MEL-5	HiSeq 2000	101	64773593	44413872
49	ENCFF028JQU	nucleus	HT1080	HiSeq 2000	101	99993883	79619839
50	ENCFF365ZYO	nucleus	HT1080	HiSeq 2000	101	100423271	78725737
51	ENCFF960DLP	cytosol	SK-N-DZ	HiSeq 2000	101	74869594	46611963
52	ENCFF138HQU	cytosol	SK-N-DZ	HiSeq 2000	101	89008399	69376251
53	ENCFF000GTX	cytosol	endothelial cell	Illumina Genome Analyzer IIx	76	115934436	100273153
54	ENCFF000GUJ	cytosol	endothelial cell	Illumina Genome Analyzer IIx	76	114090474	98916910
55	ENCFF000FAN	nucleus	GM12878	Illumina Genome Analyzer IIx	76	110469791	32504010
56	ENCFF000FAP	nucleus	GM12878	Illumina Genome Analyzer IIx	76	106919251	25485455
57	ENCFF000FPW	cytosol	HeLa-S3	Illumina Genome Analyzer IIx	76	76022570	17338563
58	ENCFF000FJF	nucleus	H1-hESC	Illumina Genome Analyzer IIx	76	104057258	67183767

59	ENCFF000HSB	cytosol	MCF-7	HiSeq 2000	101	164845832	146992074
60	ENCFF000HSC	cytosol	MCF-7	HiSeq 2000	101	157417872	141798260
61	ENCFF000HJF	cytosol	K562	Illumina Genome Analyzer IIx	76	88445339	76939567
62	ENCFF000HJP	cytosol	K562	Illumina Genome Analyzer IIx	76	124826068	106437379
63	ENCFF000FWL	cytosol	HepG2	Illumina Genome Analyzer IIx	76	111676893	21453680
64	ENCFF000FWM	cytosol	HepG2	Illumina Genome Analyzer IIx	76	128706378	25221917
65	ENCFF000INM	cytosol	SK-N-SH	HiSeq 2000	101	241123024	226343983
66	ENCFF000INN	cytosol	SK-N-SH	HiSeq 2000	101	217605980	202834226
67	ENCFF000HTD	nucleus	MCF-7	HiSeq 2000	101	141727117	91859094
68	ENCFF000HTE	nucleus	MCF-7	HiSeq 2000	101	148437796	95651372
69	ENCFF234ZAX	nucleus	K562	HiSeq 2000	50	24811186	13636828
70	ENCFF716GIG	nucleus	K562	HiSeq 2000	50	24756790	12750860
71	ENCFF000GWF	nucleus	endothelial cell	Illumina Genome Analyzer IIx	76	118116214	71462667
72	ENCFF000GWS	nucleus	endothelial cell	Illumina Genome Analyzer IIx	76	111653062	66398661
73	ENCFF000HHN	cytosol	K562	Illumina Genome Analyzer IIx	76	82063781	11183639
74	ENCFF000HIQ	cytosol	K562	Illumina Genome Analyzer IIx	76	113048736	15980585
75	ENCFF000FIM	nucleus	H1-hESC	Illumina Genome Analyzer IIx	76	93204788	22418480
76	ENCFF000IFF	nucleus	keratinocyte	Illumina Genome Analyzer IIx	76	103912304	74910537
77	ENCFF000IFI	nucleus	keratinocyte	Illumina Genome Analyzer IIx	76	120039465	88524328
78	ENCFF000IDO	nucleus	keratinocyte	Illumina Genome Analyzer IIx	76	77685202	28139399
79	ENCFF000IEM	nucleus	keratinocyte	Illumina Genome Analyzer IIx	76	105246653	31596436
80	ENCFF000HML	nucleus	K562	Illumina Genome Analyzer IIx	76	114257021	37751638
81	ENCFF000HMQ	nucleus	K562	Illumina Genome Analyzer IIx	76	92243881	32855196
82	ENCFF000FYM	nucleus	HepG2	Illumina Genome Analyzer IIx	76	117884912	36143249
83	ENCFF000FZL	nucleus	HepG2	Illumina Genome Analyzer IIx	76	20856805	6021923
84	ENCFF366XGE	nucleus	K562	HiSeq 2000	50	45511063	31000444
85	ENCFF261SZR	nucleus	K562	HiSeq 2000	50	10493637	7802327
86	ENCFF378KUR	cytosol	K562	HiSeq 2000	50	22700706	19608468
87	ENCFF763PZL	cytosol	K562	HiSeq 2000	50	29811993	25784446
88	ENCFF802YBN	nucleus	HepG2	HiSeq 2000	50	35747174	16135407
89	ENCFF417ZEN	nucleus	HepG2	HiSeq 2000	50	37390540	17165750
90	ENCFF000GUP	nucleus	endothelial cell	Illumina Genome Analyzer IIx	76	106697305	24614258
91	ENCFF000GVT	nucleus	endothelial cell	Illumina Genome Analyzer IIx	76	125001968	28502036
92	ENCFF000GTI	cytosol	endothelial cell	Illumina Genome Analyzer IIx	76	87342333	13603461
93	ENCFF000FHG	cytosol	H1-hESC	Illumina Genome Analyzer IIx	76	97222998	79797054

Table S1. RNA-Seq Sample Information.

Each RNA-Seq sample used in this study is represented with a unique ENCODE sample identifier along with their subcellular fraction, cell type, RNA sequencing platform and library read length. In addition, we display the total number of RNA-seq reads and the number of reads aligned to the transcriptome.

Feature Importance

Rank	Feature	Importance Relative	Importance Percentage
1	sense	1	0.00211168
2	TATTG	0.8002402	0.00168985
3	antisense	0.7494451	0.00158259
4	TGGAC	0.6952518	0.00146815
5	CAGGT	0.653703	0.00138041
6	TGATC	0.6098675	0.00128785
7	TGACC	0.5987518	0.00126437
8	TATTC	0.5711535	0.00120609
9	CAACT	0.5518031	0.00116523
10	TGGTG	0.5477295	0.00115663
11	TAAGT	0.5449759	0.00115082
12	AACTG	0.5416166	0.00114372
13	TAGCA	0.5400916	0.0011405
14	TTGGG	0.5383338	0.00113679
15	CTATT	0.5354362	0.00113067
16	AGCCT	0.5263705	0.00111153
17	TCTCA	0.5217201	0.00110171
18	ACTTC	0.5215629	0.00110137
19	lincRNA	0.5168682	0.00109146
20	128	0.5156315	0.00108885
21	GTAAT	0.5141062	0.00108563
22	GGGAT	0.5138292	0.00108504
23	227	0.5092402	0.00107535
24	ATTGT	0.5053074	0.00106705
25	TTACC	0.5029206	0.00106201
26	TGAAT	0.491721	0.00103836
27	ACTGA	0.49026	0.00103527
28	GATTG	0.4867606	0.00102788
29	ATGGT	0.4859861	0.00102625
30	ACCTA	0.4817451	0.00101729
31	168	0.4815713	0.00101693
32	TCTAG	0.4806321	0.00101494
33	145	0.4805863	0.00101485
34	AGGTA	0.4801835	0.00101399
35	AGTGC	0.4759634	0.00100508
36	AAGGT	0.4749889	0.00100303
37	AAAGG	0.4727931	0.00099839
38	GCAAA	0.4715982	0.00099587
39	GTGTT	0.4642348	0.00098032
40	TTTAG	0.4625999	0.00097686
41	233	0.4600181	0.00097141
42	AATCA	0.45936	0.00097002
43	GTGAG	0.4591788	0.00096964

Summary Statistics

Average relative importance of feature set

Kmer_Avg	RBP_Avg	Genomic_Avg
0.302388	0.271261	0.343282319

Percentage of variable importance of feature set

Kmer_Sum	RBP_Sum	Genomic_Sum
89.99011	8.625179	1.384713847

44	ATATC	0.4590581	0.00096938
45	GTAAG	0.4588491	0.00096894
46	GTCCA	0.458066	0.00096729
47	GTCTA	0.4565983	0.00096419
48	AAGAG	0.4543789	0.0009595
49	TTGGC	0.4528163	0.0009562
50	CAAGA	0.4518424	0.00095415
51	CTTAC	0.4512198	0.00095283
52	CCTAC	0.4499897	0.00095024
53	TTATA	0.4497904	0.00094981
54	chromosor	0.4486618	0.00094743
55	GTGAC	0.4480388	0.00094612
56	AATGT	0.4480256	0.00094609
57	GCTAT	0.4475935	0.00094518
58	GGAAT	0.4475864	0.00094516
59	CGCTA	0.4465972	0.00094307
60	GTGAA	0.4443506	0.00093833
61	GATCT	0.4426149	0.00093466
62	CCTTA	0.4414942	0.0009323
63	GAATC	0.4412491	0.00093178
64	ACAAT	0.4406241	0.00093046
65	ATGGG	0.4400584	0.00092926
66	215	0.4385456	0.00092607
67	GGCTA	0.4370823	0.00092298
68	CATGA	0.4368884	0.00092257
69	ATTCC	0.4366022	0.00092196
70	CAGAT	0.4355371	0.00091972
71	TCGAG	0.4354565	0.00091955
72	GTACA	0.4346256	0.00091779
73	GTTGT	0.4339029	0.00091626
74	GATAG	0.4327452	0.00091382
75	ATATG	0.4313167	0.0009108
76	TGCAA	0.4307379	0.00090958
77	AATTC	0.4295953	0.00090717
78	GGTAT	0.4294536	0.00090687
79	CCAAT	0.429451	0.00090686
80	CTATG	0.4282829	0.0009044
81	TAGGG	0.4282553	0.00090434
82	ATGTC	0.427979	0.00090376
83	TATGA	0.4276774	0.00090312
84	TAGGC	0.4255414	0.00089861
85	217	0.4251651	0.00089781
86	TAAGC	0.4241357	0.00089564
87	CTAAG	0.4227868	0.00089279
88	114	0.4221776	0.0008915
89	GACTA	0.421376	0.00088981
90	CTTAA	0.4202485	0.00088743

91	CAAGG	0.4202224	0.00088738
92	ATGAC	0.4198392	0.00088657
93	GATGC	0.4193126	0.00088545
94	88	0.4191133	0.00088503
95	TAGTC	0.4180542	0.0008828
96	CTAAT	0.4174441	0.00088151
97	GATTA	0.417017	0.00088061
98	AAGTC	0.4170105	0.00088059
99	ATACC	0.416658	0.00087985
100	TCTGC	0.4166486	0.00087983
101	CTGTT	0.416621	0.00087977
102	AAATC	0.4159158	0.00087828
103	GTTGA	0.4156338	0.00087769
104	TGGA	0.4141232	0.0008745
105	ATTTG	0.4136501	0.0008735
106	CACCC	0.4128258	0.00087176
107	AGGTC	0.4128082	0.00087172
108	GATGT	0.4124774	0.00087102
109	CGAAG	0.4116491	0.00086927
110	TTACT	0.4112612	0.00086845
111	GAGTT	0.4105174	0.00086688
112	CTGGG	0.4101337	0.00086607
113	AGGAC	0.4090573	0.0008638
114	TTTTA	0.4082112	0.00086201
115	GTTCT	0.4081969	0.00086198
116	TAGTT	0.407329	0.00086015
117	GCATC	0.4072979	0.00086008
118	CACTT	0.4069371	0.00085932
119	GTTAT	0.4064511	0.0008583
120	TTGCA	0.4061869	0.00085774
121	GCACT	0.4059577	0.00085725
122	TGGT	0.4054059	0.00085609
123	GGTAA	0.4053101	0.00085589
124	AGAGC	0.4051429	0.00085553
125	GACCT	0.4045441	0.00085427
126	12	0.404277	0.0008537
127	TATAG	0.4042158	0.00085358
128	TGGGA	0.4037958	0.00085269
129	CTGAT	0.4035572	0.00085218
130	TGTTA	0.4029225	0.00085084
131	CCGAC	0.4011908	0.00084719
132	44	0.4010804	0.00084695
133	ACCAC	0.4010199	0.00084683
134	GTACT	0.4009901	0.00084676
135	CAATG	0.4007503	0.00084626
136	ATTGG	0.4003891	0.00084549
137	chromosor	0.4003745	0.00084546

138	CTTGT	0.3993398	0.00084328
139	ATACT	0.3988304	0.0008422
140	CTTGC	0.3981678	0.0008408
141	AACAA	0.3979353	0.00084031
142	GTAGT	0.3975291	0.00083946
143	CTAGG	0.3968075	0.00083793
144	GGTGA	0.3965823	0.00083746
145	GGGA	0.3959783	0.00083618
146	GTCAT	0.3955594	0.0008353
147	CCGTA	0.3953662	0.00083489
148	TTTTC	0.3947403	0.00083357
149	CGGTG	0.3938605	0.00083171
150	CGTAT	0.3937843	0.00083155
151	231	0.3932822	0.00083049
152	GAGCA	0.392892	0.00082966
153	GGATG	0.3926206	0.00082909
154	GTTCC	0.3922784	0.00082837
155	TTGGA	0.3920853	0.00082796
156	GGACG	0.3916995	0.00082714
157	31	0.3908718	0.0008254
158	CTGAA	0.3908052	0.00082526
159	GGACT	0.3899935	0.00082354
160	AGGTT	0.3898897	0.00082332
161	TTTGG	0.389113	0.00082168
162	TTTAC	0.3890036	0.00082145
163	TAACA	0.3876612	0.00081862
164	CCGGA	0.3875451	0.00081837
165	CCCGG	0.3865905	0.00081636
166	AGGCA	0.3865379	0.00081625
167	CAACC	0.3864304	0.00081602
168	ACCTG	0.3861948	0.00081552
169	AGTCC	0.3860151	0.00081514
170	GTTTA	0.3858443	0.00081478
171	AAACT	0.3858052	0.0008147
172	TGGGG	0.3855855	0.00081423
173	AGCGC	0.3854426	0.00081393
174	CTGGA	0.3851914	0.0008134
175	AGATC	0.3851717	0.00081336
176	ATGCA	0.3848628	0.00081271
177	TAGTG	0.3845574	0.00081206
178	TACCT	0.3844754	0.00081189
179	GAAGA	0.384434	0.0008118
180	TATGG	0.384365	0.00081166
181	GGGG	0.3824555	0.00080762
182	CCCCA	0.382445	0.0008076
183	GAAAC	0.3820172	0.0008067
184	CCAGT	0.3818533	0.00080635

185	AAACA	0.3817826	0.0008062
186	CAGGA	0.3817631	0.00080616
187	GTTGC	0.3806855	0.00080389
188	AAACG	0.3801667	0.00080279
189	121	0.3798912	0.00080221
190	chromosor	0.3797522	0.00080192
191	GCGAA	0.3796812	0.00080177
192	GTTTG	0.3795952	0.00080158
193	GTGGT	0.37946	0.0008013
194	AGATG	0.3794336	0.00080124
195	TAACC	0.3793247	0.00080101
196	TCTTA	0.379279	0.00080092
197	GTTCA	0.3790389	0.00080041
198	AGGAA	0.3772452	0.00079662
199	ATGGA	0.37707	0.00079625
200	CAAGT	0.3767416	0.00079556
201	ATCGG	0.3766806	0.00079543
202	GAACC	0.376611	0.00079528
203	GTCAG	0.3763523	0.00079474
204	AAATT	0.3763201	0.00079467
205	CTGCG	0.3761714	0.00079435
206	TGTTC	0.3759387	0.00079386
207	GTTAA	0.3758003	0.00079357
208	TGGGT	0.3757883	0.00079355
209	TCCGG	0.3755125	0.00079296
210	GATCA	0.3752958	0.00079251
211	TAGAC	0.3747621	0.00079138
212	GAGTA	0.3744048	0.00079062
213	TCAGA	0.3738021	0.00078935
214	TGAGT	0.3737771	0.0007893
215	177	0.3736287	0.00078899
216	GCGTA	0.3729162	0.00078748
217	ACCTT	0.3728284	0.0007873
218	CAGCG	0.3727908	0.00078722
219	CTTTC	0.3726144	0.00078684
220	CTACC	0.372492	0.00078658
221	TTGCC	0.3724752	0.00078655
222	AACGT	0.3704315	0.00078223
223	TTCAT	0.3700999	0.00078153
224	AGCTA	0.3698042	0.00078091
225	GTTTC	0.3695456	0.00078036
226	CTGAG	0.3695205	0.00078031
227	CTTGA	0.3693693	0.00077999
228	CTGTA	0.3690699	0.00077936
229	CCTAA	0.3689433	0.00077909
230	AACT	0.3677116	0.00077649
231	CAACG	0.3675731	0.0007762

232	78	0.3672575	0.00077553
233	TTAGG	0.3672051	0.00077542
234	GAGC	0.3669539	0.00077489
235	GAGCG	0.3669158	0.00077481
236	CTGCT	0.3668576	0.00077469
237	GTGCC	0.3662523	0.00077341
238	ATAGA	0.3657343	0.00077231
239	CCACT	0.3653657	0.00077154
240	ACCGA	0.3645891	0.0007699
241	AAGTA	0.3642159	0.00076911
242	ATTAC	0.3639661	0.00076858
243	CGAAC	0.3637459	0.00076812
244	TTAGC	0.3636286	0.00076787
245	46	0.363493	0.00076758
246	104	0.3631357	0.00076683
247	183	0.3630118	0.00076657
248	TAAGG	0.3629938	0.00076653
249	TGCTA	0.3629641	0.00076646
250	CTATA	0.3627819	0.00076608
251	TGGTA	0.3624936	0.00076547
252	TAGCG	0.3623254	0.00076512
253	TCCTG	0.3622739	0.00076501
254	AATTG	0.3622364	0.00076493
255	TTTCC	0.3622157	0.00076488
256	225	0.3621747	0.0007648
257	GATC	0.3621617	0.00076477
258	GTAG	0.3617354	0.00076387
259	GGTTG	0.3616854	0.00076376
260	ATGAG	0.3616618	0.00076371
261	TCTGT	0.3615825	0.00076355
262	TACTC	0.3615624	0.0007635
263	TACCA	0.3614418	0.00076325
264	CAGG	0.3613884	0.00076314
265	AACAC	0.3612019	0.00076274
266	ATGCG	0.3609027	0.00076211
267	TGCTC	0.3605337	0.00076133
268	CGCCG	0.3603268	0.0007609
269	GCGCA	0.3602016	0.00076063
270	TCCGC	0.3585255	0.00075709
271	TCCAG	0.3582057	0.00075642
272	GACAG	0.3581651	0.00075633
273	AGTAG	0.3581398	0.00075628
274	AGTTC	0.3578731	0.00075571
275	TTCAG	0.3577168	0.00075538
276	ATAGG	0.3576689	0.00075528
277	ATAGT	0.3573306	0.00075457
278	CTCTC	0.3572446	0.00075439

279	GCAAT	0.3572368	0.00075437
280	TCGCT	0.3570222	0.00075392
281	CATGG	0.3568919	0.00075364
282	GGACA	0.3568098	0.00075347
283	ATGGC	0.356568	0.00075296
284	212	0.3563987	0.0007526
285	TCGGA	0.3562403	0.00075227
286	TCTTG	0.3557825	0.0007513
287	CTCCC	0.3556833	0.00075109
288	ACTTT	0.3556565	0.00075103
289	211	0.3555999	0.00075091
290	GCTAA	0.3555475	0.0007508
291	GGAAC	0.3554654	0.00075063
292	ATAAA	0.355456	0.00075061
293	GAGAC	0.3552535	0.00075018
294	CGCGG	0.355239	0.00075015
295	AGTG	0.355046	0.00074974
296	CAGCT	0.3549671	0.00074958
297	GGATA	0.3549105	0.00074946
298	TCCGT	0.3548923	0.00074942
299	GAGTG	0.3548408	0.00074931
300	AATAC	0.3547746	0.00074917
301	ATCCT	0.3547711	0.00074916
302	TAATC	0.3546522	0.00074891
303	TGTAG	0.3544554	0.0007485
304	CAGTT	0.353731	0.00074697
305	CAGCA	0.3536748	0.00074685
306	GGAGT	0.353543	0.00074657
307	TGTCA	0.3534038	0.00074628
308	GCAAC	0.3528083	0.00074502
309	TTCTT	0.3527409	0.00074488
310	TCTC	0.3524151	0.00074419
311	62	0.3514058	0.00074206
312	GGGTG	0.3513934	0.00074203
313	CTACT	0.3511795	0.00074158
314	CCTAG	0.3508191	0.00074082
315	GCTGC	0.3506168	0.00074039
316	TACTG	0.3504169	0.00073997
317	CGTCT	0.3503103	0.00073974
318	CAATA	0.3498492	0.00073877
319	CGAA	0.3498164	0.0007387
320	TTCTC	0.3497196	0.0007385
321	ATGTG	0.3495577	0.00073815
322	TGCCG	0.3494983	0.00073803
323	TTGAT	0.3493283	0.00073767
324	ACCCT	0.3489367	0.00073684
325	ATGAA	0.3488159	0.00073659

326	GCTCA	0.348767	0.00073648
327	TTCAC	0.3484101	0.00073573
328	TATC	0.3482198	0.00073533
329	CTTAT	0.3481098	0.0007351
330	CTAGC	0.3480785	0.00073503
331	ACTAA	0.3479813	0.00073483
332	CTAAA	0.3479193	0.0007347
333	GACAT	0.3474993	0.00073381
334	TGCT	0.3473203	0.00073343
335	CATG	0.3472076	0.00073319
336	AACTC	0.3469903	0.00073273
337	CACGT	0.3467658	0.00073226
338	GACAA	0.346753	0.00073223
339	GGAGC	0.3467267	0.00073218
340	CCCGA	0.3464153	0.00073152
341	166	0.3459307	0.0007305
342	GCGGA	0.3456617	0.00072993
343	CTAGA	0.3456239	0.00072985
344	CTGCA	0.345001	0.00072853
345	GAGGC	0.344619	0.00072773
346	CAACA	0.3445531	0.00072759
347	TCACC	0.3445363	0.00072755
348	CATC	0.3445188	0.00072751
349	CCGG	0.3443705	0.0007272
350	chromosor	0.3443227	0.0007271
351	CGCAC	0.3443146	0.00072708
352	CTCAA	0.3441869	0.00072681
353	CCGCG	0.3441062	0.00072664
354	CGATA	0.3440792	0.00072659
355	CCTAT	0.3439013	0.00072621
356	CATGC	0.3438232	0.00072605
357	CTCAT	0.34375	0.00072589
358	ACAGG	0.3437068	0.0007258
359	CCATC	0.3433254	0.00072499
360	AGAAC	0.3433043	0.00072495
361	ACGCA	0.343226	0.00072478
362	AAAGT	0.3431765	0.00072468
363	CACTG	0.3429129	0.00072412
364	CAGT	0.3428205	0.00072393
365	chromosor	0.3426468	0.00072356
366	GTCTT	0.342236	0.00072269
367	81	0.3419911	0.00072218
368	CCCAT	0.3419704	0.00072213
369	GGCAT	0.3417191	0.0007216
370	TTGGT	0.3416657	0.00072149
371	AAGCG	0.3415821	0.00072131
372	CAGGG	0.3415565	0.00072126

373	GAACG	0.3415115	0.00072116
374	AGACG	0.3409431	0.00071996
375	GGTAG	0.3409337	0.00071994
376	AGCTT	0.3408305	0.00071973
377	TCTCC	0.3405318	0.00071909
378	AACTT	0.3403856	0.00071879
379	ATTGC	0.3403725	0.00071876
380	GGTA	0.3403323	0.00071867
381	CGTGA	0.3401337	0.00071825
382	ACCCG	0.3400129	0.000718
383	GTATT	0.3399831	0.00071794
384	TGAC	0.3397145	0.00071737
385	CATCC	0.3395196	0.00071696
386	CGAGA	0.3392755	0.00071644
387	ATCTC	0.3392522	0.00071639
388	TTGTG	0.3391063	0.00071608
389	TTCCA	0.3389025	0.00071565
390	TGTCT	0.3387412	0.00071531
391	GACT	0.3387365	0.0007153
392	TTCCC	0.3386695	0.00071516
393	TCGT	0.3385426	0.00071489
394	GCTTC	0.3376962	0.00071311
395	132	0.3376945	0.0007131
396	TCCAC	0.3375993	0.0007129
397	GGGTT	0.337559	0.00071282
398	TAACT	0.3375059	0.00071271
399	TGAGC	0.3374876	0.00071267
400	TGGAT	0.3371221	0.00071189
401	CCGAA	0.3370889	0.00071182
402	ATGG	0.3370873	0.00071182
403	165	0.3370159	0.00071167
404	AAAAG	0.3369049	0.00071144
405	TGTCG	0.3366995	0.000711
406	TAGAA	0.3365498	0.00071069
407	TGAGA	0.33653	0.00071064
408	ACGGA	0.3364255	0.00071042
409	CCAAC	0.3363953	0.00071036
410	CAAGC	0.3361954	0.00070994
411	chromosor	0.3359783	0.00070948
412	CAGTG	0.3352227	0.00070788
413	TCAC	0.3351307	0.00070769
414	TGAAG	0.3348743	0.00070715
415	226	0.3344438	0.00070624
416	GAAGT	0.3344046	0.00070616
417	AAGAA	0.3342955	0.00070593
418	GTAGC	0.3336267	0.00070451
419	TACCG	0.3333452	0.00070392

420	GTATA	0.3333353	0.0007039
421	AACCA	0.3333131	0.00070385
422	CTTTT	0.333279	0.00070378
423	GCTGT	0.3330652	0.00070333
424	GATAT	0.3330354	0.00070327
425	CCATG	0.3329055	0.00070299
426	AATGG	0.3328951	0.00070297
427	TATCA	0.3328748	0.00070293
428	CGTGG	0.3327262	0.00070261
429	TGGCC	0.3325229	0.00070218
430	TACGA	0.3324424	0.00070201
431	ACGG	0.3324284	0.00070198
432	CGGAG	0.3319609	0.000701
433	GTCGT	0.3319488	0.00070097
434	ACGTT	0.3317389	0.00070053
435	GGCTC	0.3317123	0.00070047
436	GGAC	0.3316177	0.00070027
437	CATCA	0.3313321	0.00069967
438	TGGAA	0.3312551	0.00069951
439	ATCTA	0.3312472	0.00069949
440	AAGGG	0.3311943	0.00069938
441	AGCGT	0.330261	0.00069741
442	chromosor	0.3302062	0.00069729
443	GTTGG	0.330147	0.00069717
444	GACTG	0.3296511	0.00069612
445	CGTTG	0.3296232	0.00069606
446	17	0.3296014	0.00069601
447	156	0.3292822	0.00069534
448	AGGG	0.3289337	0.0006946
449	AGTTT	0.328606	0.00069391
450	CGACT	0.3285336	0.00069376
451	GCTGG	0.3283604	0.00069339
452	GACAC	0.3283057	0.00069328
453	TCTAC	0.3281716	0.00069299
454	GAACA	0.3280933	0.00069283
455	TACAA	0.328046	0.00069273
456	TTGCT	0.3279589	0.00069254
457	CTCGT	0.3276955	0.00069199
458	TTTCA	0.3275251	0.00069163
459	GACCG	0.3271521	0.00069084
460	CACGC	0.3270471	0.00069062
461	GGTG	0.3269891	0.0006905
462	CTGGT	0.3267531	0.00069
463	CTCAG	0.3264793	0.00068942
464	GGATT	0.3264351	0.00068933
465	14	0.3262136	0.00068886
466	GCATG	0.3260449	0.0006885

467	AAGGA	0.3260112	0.00068843
468	GGTAC	0.3255031	0.00068736
469	TCTAA	0.3254441	0.00068723
470	ACCAA	0.3253635	0.00068706
471	GAAAG	0.3253193	0.00068697
472	TAGC	0.3252626	0.00068685
473	TAAAT	0.3251305	0.00068657
474	TGCCT	0.3250871	0.00068648
475	ACCAT	0.3250357	0.00068637
476	TTCGC	0.3249495	0.00068619
477	201	0.3242627	0.00068474
478	CGCGT	0.3241203	0.00068444
479	CGTAG	0.3240775	0.00068435
480	CATTC	0.3240562	0.0006843
481	TGGAG	0.3237799	0.00068372
482	ATTGA	0.3236947	0.00068354
483	AAGGC	0.3236214	0.00068339
484	GTCCT	0.3232685	0.00068264
485	AGTAT	0.3229527	0.00068197
486	ACCTC	0.3227738	0.0006816
487	GCATA	0.3227684	0.00068158
488	GTGGG	0.3225948	0.00068122
489	CCTGA	0.3223703	0.00068074
490	CCAG	0.3223207	0.00068064
491	ACTG	0.3221741	0.00068033
492	TGTGA	0.3220011	0.00067996
493	GAGAA	0.3220003	0.00067996
494	chromosor	0.3219882	0.00067994
495	CTTCT	0.3219019	0.00067975
496	TCATA	0.3217928	0.00067952
497	AGGC	0.3217859	0.00067951
498	CTTCA	0.3215696	0.00067905
499	CTCTA	0.3210666	0.00067799
500	TCGTT	0.321051	0.00067796
501	214	0.32084	0.00067751
502	GTATC	0.3207482	0.00067732
503	CGAGC	0.3206723	0.00067716
504	GTAAC	0.3206183	0.00067704
505	CCCGT	0.3203913	0.00067656
506	TCTGA	0.3203841	0.00067655
507	TAAAG	0.3202409	0.00067625
508	chromosor	0.320101	0.00067595
509	TGTAA	0.3199516	0.00067564
510	TGAAC	0.3199213	0.00067557
511	CGGGG	0.3197737	0.00067526
512	CTGAC	0.3197684	0.00067525
513	TAAAC	0.3197142	0.00067513

514	AACGC	0.3196238	0.00067494
515	AAGAC	0.3189278	0.00067347
516	GGG	0.3186217	0.00067283
517	GAC	0.3185417	0.00067266
518	92	0.3183961	0.00067235
519	ATCTT	0.3183849	0.00067233
520	AATCC	0.3180645	0.00067165
521	CTAAC	0.3178421	0.00067118
522	chromosor	0.3178297	0.00067116
523	GCCAA	0.3176836	0.00067085
524	GGTCT	0.3176718	0.00067082
525	CTGA	0.3176577	0.00067079
526	CAGAC	0.3176188	0.00067071
527	AAAAC	0.3175576	0.00067058
528	ACCCA	0.3174818	0.00067042
529	ACATT	0.3174431	0.00067034
530	TCAGG	0.3174259	0.0006703
531	ACGAG	0.3173632	0.00067017
532	CTAGT	0.3172359	0.0006699
533	CAGAA	0.317083	0.00066958
534	GCTCT	0.3170347	0.00066948
535	GCGGC	0.3169309	0.00066926
536	GACC	0.3167404	0.00066886
537	TGTGG	0.3166665	0.0006687
538	TGCAT	0.3166632	0.00066869
539	CACCA	0.3165689	0.00066849
540	ATCAA	0.3163929	0.00066812
541	chromosor	0.3160231	0.00066734
542	GCGTT	0.3160086	0.00066731
543	ACCGG	0.3158307	0.00066693
544	ATCGC	0.3158222	0.00066692
545	CTCTT	0.3157643	0.00066679
546	AGCAT	0.3156784	0.00066661
547	TAAGA	0.3156109	0.00066647
548	3	0.3155559	0.00066635
549	TCAAG	0.3154157	0.00066606
550	CATAC	0.3153801	0.00066598
551	GTAC	0.3153007	0.00066581
552	CCTCA	0.3149973	0.00066517
553	CTACG	0.3146489	0.00066444
554	AATAT	0.3144257	0.00066397
555	CCCTT	0.3138787	0.00066281
556	ACACT	0.3137591	0.00066256
557	GC_conter	0.3136984	0.00066243
558	CGACG	0.3132865	0.00066156
559	CTCCA	0.3132139	0.00066141
560	CTTTA	0.3131269	0.00066122

561	CGCCT	0.3127519	0.00066043
562	TTACG	0.3127326	0.00066039
563	232	0.3126913	0.0006603
564	TGGG	0.3126777	0.00066028
565	CCACC	0.3123779	0.00065964
566	ACTAC	0.3122766	0.00065943
567	GTGTG	0.3122508	0.00065937
568	ATGCT	0.3121457	0.00065915
569	GAAAT	0.3119754	0.00065879
570	CCGGC	0.3115956	0.00065799
571	CCGCT	0.3115926	0.00065798
572	TCATC	0.3115866	0.00065797
573	AGCAG	0.3111391	0.00065703
574	CGATT	0.31104	0.00065682
575	133	0.3108631	0.00065644
576	71	0.3106494	0.00065599
577	CGTGC	0.3106335	0.00065596
578	GCAGA	0.3105059	0.00065569
579	GCTAG	0.31036	0.00065538
580	CTGC	0.3103029	0.00065526
581	GTAA	0.310227	0.0006551
582	CGTAA	0.3102017	0.00065505
583	CGCG	0.3100986	0.00065483
584	72	0.3097433	0.00065408
585	GGTTT	0.3097406	0.00065407
586	TTGCG	0.3096386	0.00065386
587	AGAAT	0.3093394	0.00065323
588	GTGCA	0.3093359	0.00065322
589	AAGC	0.3091382	0.0006528
590	CTCGC	0.3090953	0.00065271
591	GCCAG	0.3087839	0.00065205
592	TTCCG	0.3087305	0.00065194
593	TTAAA	0.3087113	0.0006519
594	GATTC	0.3087106	0.0006519
595	CACAT	0.3086081	0.00065168
596	ACGCG	0.3084514	0.00065135
597	CCATA	0.3082884	0.00065101
598	AGTAC	0.3081762	0.00065077
599	ACGC	0.308085	0.00065058
600	TAAC	0.3080777	0.00065056
601	AAGAT	0.3080187	0.00065044
602	GTGGC	0.3079758	0.00065035
603	AGGA	0.3074313	0.0006492
604	GACCC	0.3074092	0.00064915
605	AGAAA	0.3073989	0.00064913
606	33	0.3073542	0.00064903
607	GGCTG	0.3072971	0.00064891

608	CATAA	0.3072855	0.00064889
609	CATGT	0.3072566	0.00064883
610	TTTGC	0.3067971	0.00064786
611	GCCG	0.3067818	0.00064783
612	CGGCG	0.3066428	0.00064753
613	ATCCC	0.3066384	0.00064752
614	GCATT	0.3066049	0.00064745
615	GTCTG	0.3064152	0.00064705
616	CATTA	0.3062476	0.0006467
617	AGCTG	0.3061681	0.00064653
618	TACAG	0.3061384	0.00064647
619	GGTCA	0.3060377	0.00064625
620	ACCAG	0.3060117	0.0006462
621	CCAGG	0.3058646	0.00064589
622	ACAA	0.3057863	0.00064572
623	TCGAT	0.3057852	0.00064572
624	CAAAC	0.3057234	0.00064559
625	TATAC	0.3057122	0.00064557
626	CGGCT	0.3054993	0.00064512
627	135	0.305319	0.00064474
628	AGGAT	0.3051075	0.00064429
629	GAACT	0.3050987	0.00064427
630	CCCCC	0.3048911	0.00064383
631	167	0.3048252	0.00064369
632	TACGT	0.3045697	0.00064315
633	CCATT	0.3042824	0.00064255
634	AGTCT	0.3042196	0.00064242
635	83	0.3041429	0.00064225
636	GATTT	0.3040887	0.00064214
637	TCG	0.3040685	0.0006421
638	TTTTG	0.3040269	0.00064201
639	TCCAT	0.3040158	0.00064198
640	TTTCT	0.303967	0.00064188
641	GGGCC	0.3036401	0.00064119
642	AGCAA	0.3035835	0.00064107
643	CGG	0.3035707	0.00064104
644	GGGGA	0.3035556	0.00064101
645	AGCCG	0.3034559	0.0006408
646	AATCT	0.3032231	0.00064031
647	CCAC	0.3030256	0.00063989
648	101	0.3029267	0.00063968
649	CTAA	0.3029061	0.00063964
650	AGTTG	0.3027285	0.00063927
651	CGTA	0.302526	0.00063884
652	GTGTC	0.3023171	0.0006384
653	TGTTG	0.3023168	0.0006384
654	CGAAT	0.3021215	0.00063798

655	ACGGT	0.3020908	0.00063792
656	208	0.3018283	0.00063737
657	GTCG	0.3017987	0.0006373
658	200	0.3016985	0.00063709
659	TGGCG	0.3016666	0.00063702
660	CACGG	0.3014509	0.00063657
661	TAGGA	0.3013919	0.00063644
662	ATTAT	0.3013901	0.00063644
663	TCACG	0.3012852	0.00063622
664	CAATC	0.3011758	0.00063599
665	CGTTT	0.3011606	0.00063596
666	ATCAC	0.3011161	0.00063586
667	GAGTC	0.3010118	0.00063564
668	ACTCT	0.3009234	0.00063545
669	AACAT	0.3008593	0.00063532
670	ATGTT	0.3007143	0.00063501
671	CGTAC	0.30056	0.00063469
672	AAAAA	0.3004104	0.00063437
673	CGCAT	0.3003182	0.00063418
674	GGTTA	0.3003096	0.00063416
675	CAGAG	0.2999154	0.00063333
676	AGCCC	0.2998483	0.00063318
677	CGCAG	0.2996723	0.00063281
678	CGTTA	0.2995442	0.00063254
679	ATCG	0.2994582	0.00063236
680	GAAG	0.2993654	0.00063216
681	CTCCT	0.2993059	0.00063204
682	GAAGC	0.2990652	0.00063153
683	CTCT	0.2989322	0.00063125
684	TACTT	0.2989084	0.0006312
685	CGGGT	0.29882	0.00063101
686	AATTT	0.2988116	0.000631
687	AGACT	0.2987623	0.00063089
688	TAGTA	0.2984904	0.00063032
689	191	0.2983585	0.00063004
690	GGATC	0.2982444	0.0006298
691	TGCTG	0.2982065	0.00062972
692	ACGGC	0.2981231	0.00062954
693	GGTTC	0.2981176	0.00062953
694	GGCGT	0.2980856	0.00062946
695	GGTCG	0.2979112	0.00062909
696	ATGTA	0.2978418	0.00062895
697	ACTCA	0.2977764	0.00062881
698	24	0.2977435	0.00062874
699	TTGTA	0.2976995	0.00062865
700	AGTCG	0.2976087	0.00062846
701	103	0.2975773	0.00062839

702	AGAGT	0.2975149	0.00062826
703	CTATC	0.2973515	0.00062791
704	ATGCC	0.2971194	0.00062742
705	TGAG	0.2970482	0.00062727
706	4	0.2970007	0.00062717
707	TCCTC	0.29685	0.00062685
708	AGGCT	0.2967876	0.00062672
709	chromosor	0.2967611	0.00062667
710	ACGTC	0.2966399	0.00062641
711	TCAAC	0.2966195	0.00062637
712	AGTGT	0.2964055	0.00062591
713	AGAGG	0.2963185	0.00062573
714	ATCAG	0.2963099	0.00062571
715	ATAAG	0.2962935	0.00062568
716	ACAAG	0.2962736	0.00062564
717	GCTTG	0.2961135	0.0006253
718	TAGCC	0.2958053	0.00062465
719	GTGTA	0.2956346	0.00062429
720	CGATC	0.2955892	0.00062419
721	GCCGG	0.2955082	0.00062402
722	GTAGA	0.2954646	0.00062393
723	AGGT	0.2954101	0.00062381
724	AAGCC	0.2953011	0.00062358
725	CGA	0.2950472	0.00062305
726	AATGC	0.2945846	0.00062207
727	TTAGA	0.2945693	0.00062204
728	ATTAG	0.2942237	0.00062131
729	GGGTA	0.2941374	0.00062112
730	TAACG	0.2941103	0.00062107
731	CAAA	0.2941001	0.00062105
732	CATTT	0.2936741	0.00062015
733	GCCGC	0.293667	0.00062013
734	AAGA	0.2935825	0.00061995
735	TCAAT	0.2934653	0.00061971
736	TCGTG	0.2933593	0.00061948
737	AGTGA	0.2932439	0.00061924
738	ACCGT	0.2932207	0.00061919
739	AGCAC	0.2931307	0.000619
740	GCTGA	0.2930979	0.00061893
741	GACGC	0.2928782	0.00061847
742	chromosor	0.2928452	0.0006184
743	AATAG	0.2927526	0.0006182
744	CGCTT	0.2927525	0.0006182
745	152	0.2927208	0.00061813
746	TGATG	0.2926546	0.00061799
747	TGGCA	0.292375	0.0006174
748	AACCC	0.2922436	0.00061713

749	CTACA	0.2922435	0.00061713
750	GCACC	0.292064	0.00061675
751	TCCAA	0.2920312	0.00061668
752	ACAGT	0.2920238	0.00061666
753	TCAGT	0.2919892	0.00061659
754	AACTA	0.2919563	0.00061652
755	TACGC	0.2918374	0.00061627
756	GGCTT	0.2916953	0.00061597
757	CCACA	0.2915873	0.00061574
758	TAGA	0.2915477	0.00061566
759	AGAAG	0.2915166	0.00061559
760	GGGAC	0.2914963	0.00061555
761	AACCG	0.2913996	0.00061534
762	TTCG	0.2911301	0.00061477
763	GTCCC	0.2910493	0.0006146
764	TCTG	0.2909099	0.00061431
765	ACGA	0.2908136	0.00061411
766	CGCAA	0.2908078	0.00061409
767	ATATT	0.2907855	0.00061405
768	CTAC	0.2906603	0.00061378
769	CGCC	0.2901624	0.00061273
770	TCAG	0.2900951	0.00061259
771	TGTGT	0.2899532	0.00061229
772	GCTTT	0.2898411	0.00061205
773	47	0.2897389	0.00061184
774	AGTAA	0.2897208	0.0006118
775	CGTCA	0.2891569	0.00061061
776	7	0.2890806	0.00061045
777	GCGAG	0.289039	0.00061036
778	GCGAT	0.2890108	0.0006103
779	94	0.2890033	0.00061028
780	TACA	0.2889957	0.00061027
781	TCACA	0.2887981	0.00060985
782	CCTCC	0.2887884	0.00060983
783	ACACA	0.2887179	0.00060968
784	CACAG	0.2886947	0.00060963
785	CCCAC	0.2886057	0.00060944
786	CGAGT	0.2885646	0.00060936
787	TCGA	0.2884867	0.00060919
788	GGCCG	0.2884684	0.00060915
789	GCT	0.2884453	0.0006091
790	GCTCG	0.2884415	0.0006091
791	GGGGT	0.2884114	0.00060903
792	AACGA	0.2883374	0.00060888
793	AACAG	0.2879298	0.00060802
794	CCTGG	0.2879151	0.00060799
795	AC	0.2877709	0.00060768

796	TGTCC	0.2877646	0.00060767
797	GTCTC	0.2876667	0.00060746
798	CTAG	0.2875632	0.00060724
799	CGGTT	0.2873798	0.00060685
800	AAGCA	0.2873447	0.00060678
801	GTCCG	0.2872877	0.00060666
802	TCGGG	0.287136	0.00060634
803	ATCCG	0.2871041	0.00060627
804	8	0.2870916	0.00060625
805	87	0.2870755	0.00060621
806	GGGAG	0.2870726	0.00060621
807	GCAC	0.2870073	0.00060607
808	TCGTA	0.2868711	0.00060578
809	CGGAC	0.2868442	0.00060572
810	TATCT	0.2868169	0.00060567
811	GGGCT	0.2866482	0.00060531
812	ACATA	0.2863418	0.00060466
813	AGTCA	0.286189	0.00060434
814	GTTAG	0.2861203	0.0006042
815	CG	0.2860618	0.00060407
816	ATTTA	0.2857563	0.00060343
817	chromosor	0.2853747	0.00060262
818	CAAAG	0.2853226	0.00060251
819	AGTTA	0.2852818	0.00060242
820	GTAGG	0.2849953	0.00060182
821	CCCCG	0.2849286	0.00060168
822	ACAAA	0.2846316	0.00060105
823	ACCGC	0.284499	0.00060077
824	ACG	0.284268	0.00060028
825	TTATT	0.2841106	0.00059995
826	CAAT	0.283909	0.00059953
827	TAGCT	0.2838138	0.00059932
828	TCCCA	0.2837829	0.00059926
829	TGCGC	0.2837017	0.00059909
830	CGAAA	0.283425	0.0005985
831	9	0.2832747	0.00059819
832	TAATG	0.282966	0.00059753
833	CGAGG	0.2828196	0.00059723
834	TTACA	0.2826321	0.00059683
835	AAATG	0.2826247	0.00059681
836	GGACC	0.2824893	0.00059653
837	230	0.2824397	0.00059642
838	CATTG	0.2823587	0.00059625
839	GGAAG	0.282153	0.00059582
840	CTTTG	0.2818892	0.00059526
841	TACAT	0.2818868	0.00059526
842	AGTC	0.2818758	0.00059523

843	TGCA	0.2817059	0.00059487
844	GACGG	0.2816604	0.00059478
845	GATGG	0.2815211	0.00059448
846	AAAGA	0.2814908	0.00059442
847	ACATG	0.2811933	0.00059379
848	CTTCG	0.2810739	0.00059354
849	67	0.280818	0.000593
850	GAGGG	0.2807035	0.00059276
851	TTAAT	0.280658	0.00059266
852	CCAAA	0.2806016	0.00059254
853	TTAGT	0.2805098	0.00059235
854	ATCC	0.2804138	0.00059214
855	CAGGC	0.2804085	0.00059213
856	TCAAA	0.2804033	0.00059212
857	70	0.2798586	0.00059097
858	CTCA	0.2796205	0.00059047
859	GCCTC	0.2795714	0.00059037
860	GTAAA	0.2793093	0.00058981
861	20	0.2792943	0.00058978
862	chromosor	0.2792697	0.00058973
863	chromosor	0.2790821	0.00058933
864	TCAGC	0.2787235	0.00058858
865	CACCG	0.2786898	0.0005885
866	106	0.2786516	0.00058842
867	AATTA	0.2786253	0.00058837
868	CGTGT	0.2785546	0.00058822
869	GGAAA	0.2785523	0.00058821
870	ATCA	0.2785234	0.00058815
871	CTCCG	0.2784608	0.00058802
872	TCACT	0.2784144	0.00058792
873	TTGG	0.2783568	0.0005878
874	GG	0.2783114	0.00058771
875	GAAGG	0.2782433	0.00058756
876	ACAGC	0.2781105	0.00058728
877	TTAG	0.278102	0.00058726
878	ATAG	0.2779145	0.00058687
879	GCAGC	0.2775569	0.00058611
880	AAGT	0.2774155	0.00058581
881	TAAG	0.2773711	0.00058572
882	TAGGT	0.2773632	0.0005857
883	AGGCG	0.2773522	0.00058568
884	ACCCC	0.2771397	0.00058523
885	206	0.2770813	0.00058511
886	216	0.2768474	0.00058461
887	108	0.2768269	0.00058457
888	GCTT	0.276766	0.00058444
889	TGATA	0.2766148	0.00058412

890	TAGAG	0.2765714	0.00058403
891	GTCAC	0.2765054	0.00058389
892	ACTTG	0.2764978	0.00058388
893	TTCTG	0.2764832	0.00058384
894	GTGGA	0.2764177	0.00058371
895	TGCGT	0.2764134	0.0005837
896	TCCCG	0.276393	0.00058365
897	TCTAT	0.2762652	0.00058338
898	85	0.2762456	0.00058334
899	GGCCC	0.2757559	0.00058231
900	TGGTC	0.2756978	0.00058219
901	TG	0.2755784	0.00058193
902	ACGAA	0.2755758	0.00058193
903	GGCAC	0.2755002	0.00058177
904	TTATC	0.2753664	0.00058149
905	178	0.2753537	0.00058146
906	CGGT	0.2753294	0.00058141
907	CATAG	0.2752903	0.00058133
908	CTTCC	0.2751506	0.00058103
909	CGGTC	0.2751353	0.000581
910	TTCAA	0.2750615	0.00058084
911	CCAGA	0.2746572	0.00057999
912	TATGC	0.2745674	0.0005798
913	GTG	0.2745595	0.00057978
914	ACGAC	0.2745208	0.0005797
915	TCGTC	0.2741716	0.00057896
916	64	0.274134	0.00057888
917	AGACA	0.2739513	0.0005785
918	18	0.2738108	0.0005782
919	CCTGC	0.2737538	0.00057808
920	AGGGA	0.2736314	0.00057782
921	GTC	0.2735502	0.00057765
922	TCTTC	0.2734826	0.00057751
923	GGCAA	0.2734193	0.00057737
924	CGCCA	0.2733808	0.00057729
925	TACTA	0.273359	0.00057725
926	GGGTC	0.2733426	0.00057721
927	AGGGC	0.2732926	0.00057711
928	GCCTA	0.2732746	0.00057707
929	CCCAG	0.2730667	0.00057663
930	AGCG	0.2729649	0.00057642
931	176	0.2729029	0.00057628
932	82	0.2728448	0.00057616
933	GAGAT	0.2728187	0.00057611
934	AAACC	0.2727523	0.00057597
935	GCCAC	0.2726657	0.00057578
936	GATCC	0.2726595	0.00057577

937	TGTA	0.272621	0.00057569
938	AGAGA	0.2725977	0.00057564
939	GCTAC	0.2725891	0.00057562
940	43	0.2725878	0.00057562
941	TCGGC	0.2724377	0.0005753
942	ACACG	0.2723026	0.00057502
943	CGTCC	0.2722906	0.00057499
944	GAGCC	0.2722615	0.00057493
945	GCCCC	0.2722201	0.00057484
946	AGATA	0.2722108	0.00057482
947	93	0.2721845	0.00057477
948	CGGAA	0.2721677	0.00057473
949	GCGTG	0.2721319	0.00057466
950	ATAC	0.2721316	0.00057466
951	GGTCC	0.2721226	0.00057464
952	GCGAC	0.2719387	0.00057425
953	TTCGT	0.2718933	0.00057415
954	CTTAG	0.271842	0.00057404
955	112	0.2718162	0.00057399
956	58	0.2717783	0.00057391
957	GGAGA	0.271699	0.00057374
958	GGCCA	0.271612	0.00057356
959	127	0.2715174	0.00057336
960	146	0.2713278	0.00057296
961	TCAT	0.2713219	0.00057295
962	TTATG	0.2709638	0.00057219
963	GCGCT	0.2708451	0.00057194
964	GTGCG	0.2707382	0.00057171
965	CGCTG	0.2707109	0.00057166
966	GTGC	0.2706113	0.00057145
967	TATCC	0.2706011	0.00057142
968	CCGCA	0.2705126	0.00057124
969	TGTTT	0.2704895	0.00057119
970	TTTAA	0.2703655	0.00057093
971	TCTGG	0.2703441	0.00057088
972	55	0.2702963	0.00057078
973	GTTA	0.2701044	0.00057037
974	GACCA	0.2701023	0.00057037
975	GACTT	0.2700354	0.00057023
976	ACCG	0.2699537	0.00057006
977	GGAGG	0.2698562	0.00056985
978	TCTCG	0.2697844	0.0005697
979	GAATA	0.2697818	0.00056969
980	TCTT	0.2696791	0.00056948
981	100	0.2695808	0.00056927
982	ACAT	0.2695502	0.0005692
983	TTCC	0.2692024	0.00056847

984	AGAA	0.2691171	0.00056829
985	TGAGG	0.2690529	0.00056815
986	79	0.2689447	0.00056793
987	GCGCC	0.2689152	0.00056786
988	CGGC	0.268898	0.00056783
989	ACTGG	0.2688006	0.00056762
990	GCTCC	0.2686152	0.00056723
991	TACCC	0.2685218	0.00056703
992	CAGA	0.2684454	0.00056687
993	CCCTC	0.2684053	0.00056679
994	102	0.2682732	0.00056651
995	TATAA	0.268236	0.00056643
996	AAAT	0.2680425	0.00056602
997	GGTGC	0.2679881	0.00056591
998	GTCA	0.2679259	0.00056577
999	AACG	0.2677739	0.00056545
1000	CGGCC	0.2677712	0.00056545
1001	GAGGA	0.2675619	0.00056501
1002	2	0.2675429	0.00056497
1003	ACAC	0.2674552	0.00056478
1004	TATAT	0.2673063	0.00056447
1005	GTGAT	0.2672216	0.00056429
1006	AAT	0.2670873	0.000564
1007	ATGAT	0.2670188	0.00056386
1008	143	0.2669109	0.00056363
1009	AATCG	0.2668513	0.00056351
1010	ACTAT	0.2668132	0.00056342
1011	AGGGG	0.266806	0.00056341
1012	CGCGC	0.2667228	0.00056323
1013	TTCCT	0.2667144	0.00056322
1014	GGGT	0.2666867	0.00056316
1015	chromosor	0.2664915	0.00056275
1016	GAA	0.2663946	0.00056254
1017	CTA	0.2663898	0.00056253
1018	CACT	0.2663736	0.0005625
1019	TCCC	0.2662494	0.00056223
1020	GCAGT	0.2662353	0.0005622
1021	TCTTT	0.2661659	0.00056206
1022	ATAAT	0.2661449	0.00056201
1023	ACTC	0.2660692	0.00056185
1024	CTCTG	0.2660692	0.00056185
1025	CCTCT	0.2660586	0.00056183
1026	75	0.2659886	0.00056168
1027	CGCCC	0.2659353	0.00056157
1028	TCGCG	0.2657051	0.00056108
1029	TTCTA	0.2655693	0.0005608
1030	28	0.2655214	0.0005607

1031	GCAA	0.2654687	0.00056059
1032	CGGA	0.2652714	0.00056017
1033	TCCG	0.2651451	0.0005599
1034	CCCAA	0.2650265	0.00055965
1035	ACAGA	0.2647398	0.00055905
1036	ACTCG	0.2646645	0.00055889
1037	GTTTT	0.2646028	0.00055876
1038	GTATG	0.2645258	0.00055859
1039	49	0.2644053	0.00055834
1040	CAGTC	0.2643496	0.00055822
1041	6	0.2642337	0.00055798
1042	36	0.2642125	0.00055793
1043	ACAG	0.2641573	0.00055782
1044	GATT	0.2640217	0.00055753
1045	ATCT	0.2640008	0.00055749
1046	CTCC	0.2639743	0.00055743
1047	CTGTG	0.2639053	0.00055728
1048	CCTA	0.2638597	0.00055719
1049	CTCGA	0.2636375	0.00055672
1050	GACTC	0.2635834	0.0005566
1051	TGTG	0.2635818	0.0005566
1052	48	0.2635571	0.00055655
1053	TCCCC	0.2635536	0.00055654
1054	AGT	0.2634138	0.00055625
1055	CGATG	0.2632076	0.00055581
1056	chromosor	0.2631516	0.00055569
1057	TTTA	0.2631312	0.00055565
1058	141	0.2630922	0.00055557
1059	ACTAG	0.2629468	0.00055526
1060	97	0.2628913	0.00055514
1061	TCAA	0.262787	0.00055492
1062	TTGAG	0.2627072	0.00055475
1063	CAGCC	0.2626795	0.0005547
1064	GCAT	0.2625745	0.00055447
1065	CCCTA	0.2625501	0.00055442
1066	GTTC	0.2625284	0.00055438
1067	125	0.2622995	0.00055389
1068	GCTA	0.2622173	0.00055372
1069	TCA	0.262071	0.00055341
1070	CACG	0.2620187	0.0005533
1071	37	0.2619344	0.00055312
1072	203	0.2618138	0.00055287
1073	GGGCG	0.261802	0.00055284
1074	TGCAC	0.2616996	0.00055263
1075	CACAA	0.2616863	0.0005526
1076	GACGT	0.2616643	0.00055255
1077	TCGCC	0.2613157	0.00055182

1078	CCTGT	0.2611655	0.0005515
1079	AGTA	0.2607029	0.00055052
1080	chromosor	0.2605324	0.00055016
1081	GCCT	0.2604497	0.00054999
1082	TAATT	0.2603879	0.00054986
1083	ACGTG	0.2598177	0.00054865
1084	AACC	0.2597427	0.00054849
1085	30	0.2596336	0.00054826
1086	GCCGT	0.2596288	0.00054825
1087	ACCT	0.2596134	0.00054822
1088	CAG	0.2595974	0.00054819
1089	CGC	0.2595346	0.00054805
1090	ATATA	0.2593883	0.00054775
1091	GAGCT	0.2593163	0.00054759
1092	50	0.2592764	0.00054751
1093	CCGAG	0.2592059	0.00054736
1094	GCGTC	0.2590299	0.00054699
1095	TTAAG	0.2588319	0.00054657
1096	ACGTA	0.2588189	0.00054654
1097	GGGGC	0.2587395	0.00054638
1098	GCCTT	0.2587217	0.00054634
1099	184	0.2585546	0.00054599
1100	TTAA	0.2585022	0.00054587
1101	GGGCA	0.2583568	0.00054557
1102	123	0.2583207	0.00054549
1103	GGTC	0.2583101	0.00054547
1104	GATG	0.2580614	0.00054494
1105	GGCC	0.2580357	0.00054489
1106	TATCG	0.2580264	0.00054487
1107	TATTT	0.2580186	0.00054485
1108	GCGGG	0.2579588	0.00054473
1109	TAC	0.2579206	0.00054465
1110	AAGG	0.2578968	0.0005446
1111	122	0.2577735	0.00054434
1112	AAGTG	0.2576528	0.00054408
1113	19	0.2576135	0.000544
1114	AAC	0.2575786	0.00054392
1115	chromosor	0.2575524	0.00054387
1116	GCCGA	0.2574034	0.00054355
1117	GTCAA	0.2573168	0.00054337
1118	CGCGA	0.257183	0.00054309
1119	GATGA	0.2570958	0.0005429
1120	GAAAA	0.2570766	0.00054286
1121	TGTGC	0.2569487	0.00054259
1122	ACTA	0.2569292	0.00054255
1123	AGGGT	0.2568813	0.00054245
1124	ACTGT	0.2568343	0.00054235

1125	GCCCA	0.2568085	0.0005423
1126	CTTG	0.2568079	0.0005423
1127	GGAT	0.2566254	0.00054191
1128	GGAG	0.2564914	0.00054163
1129	TCTCT	0.2562298	0.00054108
1130	CCCCT	0.2561852	0.00054098
1131	GCTC	0.256131	0.00054087
1132	AGGCC	0.2560813	0.00054076
1133	CGTC	0.2560647	0.00054073
1134	TCCGA	0.2560565	0.00054071
1135	TTGTC	0.2560561	0.00054071
1136	AAAGC	0.2560185	0.00054063
1137	GCCAT	0.2558872	0.00054035
1138	185	0.2556835	0.00053992
1139	CGCA	0.2555979	0.00053974
1140	TTAT	0.2555711	0.00053968
1141	110	0.2554549	0.00053944
1142	CCGCC	0.2554492	0.00053943
1143	CTGCC	0.255436	0.0005394
1144	TTCGA	0.2553616	0.00053924
1145	CAA	0.2552801	0.00053907
1146	GAAT	0.2552086	0.00053892
1147	41	0.2551127	0.00053872
1148	TGTT	0.2550798	0.00053865
1149	ATCTG	0.2548113	0.00053808
1150	TCGCA	0.2547235	0.0005379
1151	213	0.2546738	0.00053779
1152	10	0.2545525	0.00053753
1153	AACGG	0.2543973	0.00053721
1154	ACATC	0.2543169	0.00053704
1155	118	0.2541166	0.00053661
1156	99	0.2541142	0.00053661
1157	TAGAT	0.2540982	0.00053657
1158	CAATT	0.2540678	0.00053651
1159	GGCAG	0.2539938	0.00053635
1160	GCCCT	0.2536375	0.0005356
1161	TGTAC	0.2536294	0.00053558
1162	150	0.2536126	0.00053555
1163	TAG	0.2534497	0.00053521
1164	26	0.2529368	0.00053412
1165	TGC	0.2528569	0.00053395
1166	GGGGG	0.252756	0.00053374
1167	GATA	0.2526709	0.00053356
1168	AACCT	0.2525792	0.00053337
1169	CATA	0.2524741	0.00053315
1170	140	0.2521595	0.00053248
1171	ATTCT	0.2520655	0.00053228

1172	TCATT	0.252052	0.00053225
1173	GGCGC	0.2520425	0.00053223
1174	AAAC	0.2518934	0.00053192
1175	147	0.2518227	0.00053177
1176	CCGT	0.2517224	0.00053156
1177	CCTTC	0.2516654	0.00053144
1178	ACGT	0.2516437	0.00053139
1179	AGG	0.2514939	0.00053108
1180	ACTTA	0.251453	0.00053099
1181	GGC	0.2513652	0.0005308
1182	CTGGC	0.2513624	0.0005308
1183	TTGAC	0.2512464	0.00053055
1184	TCGGT	0.2511251	0.0005303
1185	CAGC	0.2511062	0.00053026
1186	ATAGC	0.2510837	0.00053021
1187	107	0.2509153	0.00052985
1188	GGTGT	0.2508518	0.00052972
1189	25	0.2508421	0.0005297
1190	TCCCT	0.2507566	0.00052952
1191	GCGGT	0.2506769	0.00052935
1192	204	0.2506769	0.00052935
1193	CCGTC	0.2506666	0.00052933
1194	AGGAG	0.2506498	0.00052929
1195	CGGAT	0.2505638	0.00052911
1196	GCTG	0.2495326	0.00052693
1197	TTGAA	0.2494875	0.00052684
1198	66	0.2491278	0.00052608
1199	CTC	0.2489739	0.00052575
1200	111	0.2488723	0.00052554
1201	TGGGC	0.2488438	0.00052548
1202	GGGAA	0.2486439	0.00052506
1203	GGTGG	0.2485456	0.00052485
1204	13	0.2484598	0.00052467
1205	CCCC	0.2481099	0.00052393
1206	ATTCA	0.2479363	0.00052356
1207	139	0.2478968	0.00052348
1208	39	0.2478512	0.00052338
1209	CCACG	0.2478267	0.00052333
1210	TGGCT	0.2477924	0.00052326
1211	TGTC	0.2477383	0.00052314
1212	GCCCG	0.2477155	0.0005231
1213	GAATT	0.2476471	0.00052295
1214	CGGCA	0.2475582	0.00052276
1215	GTTAC	0.2474903	0.00052262
1216	ATCCA	0.2474459	0.00052253
1217	53	0.2472256	0.00052206
1218	CGACC	0.2471552	0.00052191

1219	ACC	0.247154	0.00052191
1220	GAGGT	0.2466931	0.00052094
1221	AGATT	0.2466212	0.00052079
1222	AGCCA	0.2465344	0.0005206
1223	ATCAT	0.2465037	0.00052054
1224	CATCT	0.246345	0.0005202
1225	TCCTT	0.2460165	0.00051951
1226	ACACC	0.2459612	0.00051939
1227	AGTGG	0.2457477	0.00051894
1228	TACGG	0.2457218	0.00051889
1229	CGACA	0.2457186	0.00051888
1230	AGC	0.24571	0.00051886
1231	27	0.2455248	0.00051847
1232	202	0.2452679	0.00051793
1233	GGAA	0.2451754	0.00051773
1234	TTGT	0.2449121	0.00051718
1235	CGGTA	0.2447951	0.00051693
1236	TGATT	0.2445581	0.00051643
1237	AATG	0.2444958	0.0005163
1238	TGGTT	0.2443863	0.00051607
1239	CATAT	0.2443856	0.00051606
1240	CCAGC	0.2443175	0.00051592
1241	ACTT	0.2440839	0.00051543
1242	GTAT	0.2440657	0.00051539
1243	GAAA	0.24392	0.00051508
1244	GCACA	0.2438712	0.00051498
1245	138	0.2438426	0.00051492
1246	CACTC	0.2438191	0.00051487
1247	CCGGG	0.2436926	0.0005146
1248	ACTCC	0.2435612	0.00051432
1249	AGCC	0.2433134	0.0005138
1250	ATCGA	0.2432424	0.00051365
1251	234	0.2430153	0.00051317
1252	TTTAT	0.2429725	0.00051308
1253	CCGAT	0.242886	0.0005129
1254	AACA	0.2428007	0.00051272
1255	CACTA	0.2427561	0.00051262
1256	GCAAG	0.2426948	0.00051249
1257	GCGC	0.2426417	0.00051238
1258	ATACA	0.2423204	0.0005117
1259	ACGCT	0.2422629	0.00051158
1260	AGACC	0.242219	0.00051149
1261	CCGA	0.2419475	0.00051092
1262	CCAT	0.2419033	0.00051082
1263	CA	0.2418506	0.00051071
1264	74	0.2412135	0.00050937
1265	TCTA	0.2411868	0.00050931

1266	CCCA	0.2411426	0.00050922
1267	CTCG	0.241105	0.00050914
1268	AGAG	0.240726	0.00050834
1269	GGCGA	0.2402623	0.00050736
1270	CTTGG	0.2400546	0.00050692
1271	89	0.2396899	0.00050615
1272	CCGTT	0.2396789	0.00050613
1273	AAAG	0.2396357	0.00050603
1274	29	0.2395417	0.00050584
1275	TGAT	0.2394965	0.00050574
1276	AAGTT	0.2392407	0.0005052
1277	GTACC	0.2391997	0.00050511
1278	CCG	0.2391071	0.00050492
1279	CACAC	0.2390927	0.00050489
1280	169	0.238984	0.00050466
1281	GTT	0.2389629	0.00050461
1282	AGAC	0.2386519	0.00050396
1283	76	0.2385684	0.00050378
1284	TACG	0.2385218	0.00050368
1285	TCGC	0.2384558	0.00050354
1286	38	0.2384344	0.0005035
1287	ATTA	0.2381539	0.00050291
1288	86	0.2378726	0.00050231
1289	TGAAA	0.2378581	0.00050228
1290	AAAAT	0.2377625	0.00050208
1291	TTGTT	0.2375181	0.00050156
1292	TCT	0.237506	0.00050154
1293	GACA	0.2374307	0.00050138
1294	TGCAG	0.2373832	0.00050128
1295	AGCT	0.2373548	0.00050122
1296	ACAAC	0.2371739	0.00050084
1297	TGTAT	0.2370466	0.00050057
1298	CCGTG	0.2368951	0.00050025
1299	ATGC	0.2367512	0.00049994
1300	119	0.2366223	0.00049967
1301	AAAA	0.2364882	0.00049939
1302	GGCA	0.2362776	0.00049894
1303	TGAA	0.2360691	0.0004985
1304	TAAT	0.23597	0.00049829
1305	CCCGC	0.2358855	0.00049812
1306	GTTCG	0.2355969	0.00049751
1307	45	0.2355756	0.00049746
1308	CGAC	0.2354975	0.0004973
1309	chromosor	0.2353532	0.00049699
1310	TTTGT	0.235228	0.00049673
1311	GTTT	0.2351909	0.00049665
1312	98	0.2351556	0.00049657

1313	84	0.2349828	0.00049621
1314	GATAC	0.2348296	0.00049589
1315	TGCC	0.234789	0.0004958
1316	GCACG	0.2347783	0.00049578
1317	GCA	0.2345872	0.00049537
1318	GCG	0.2343999	0.00049498
1319	ACCA	0.2343352	0.00049484
1320	GACGA	0.23418	0.00049451
1321	TACAC	0.2341044	0.00049435
1322	CCCTG	0.2340733	0.00049429
1323	GTGA	0.2339676	0.00049407
1324	TAGT	0.2339498	0.00049403
1325	ACGAT	0.2338164	0.00049375
1326	TTA	0.2338091	0.00049373
1327	CATCG	0.2337267	0.00049356
1328	90	0.2336936	0.00049349
1329	CAGTA	0.2336775	0.00049345
1330	TGCCA	0.233345	0.00049275
1331	TAATA	0.2332796	0.00049261
1332	96	0.233105	0.00049224
1333	126	0.2327511	0.0004915
1334	GTGG	0.2323414	0.00049063
1335	GCTTA	0.2321668	0.00049026
1336	TGCCC	0.2319479	0.0004898
1337	TATT	0.2318868	0.00048967
1338	155	0.2316934	0.00048926
1339	GT	0.2316882	0.00048925
1340	CTTT	0.2316666	0.00048921
1341	GCGG	0.2316166	0.0004891
1342	CAAAT	0.2313991	0.00048864
1343	GATCG	0.2312816	0.00048839
1344	GCGCG	0.2311964	0.00048821
1345	AAA	0.2308876	0.00048756
1346	CAAC	0.2308167	0.00048741
1347	163	0.230646	0.00048705
1348	AATAA	0.2304688	0.00048668
1349	TATGT	0.2303834	0.0004865
1350	TGCTT	0.2302943	0.00048631
1351	CCA	0.230258	0.00048623
1352	GAGG	0.230006	0.0004857
1353	ATTCG	0.2298087	0.00048528
1354	TCCTA	0.2296872	0.00048503
1355	CTGG	0.229382	0.00048438
1356	TTAC	0.2293506	0.00048432
1357	TTTG	0.2291879	0.00048397
1358	TTGC	0.2291837	0.00048396
1359	GGCCT	0.2290699	0.00048372

1360	CACGA	0.2290438	0.00048367
1361	TTAAC	0.2289116	0.00048339
1362	TCCT	0.2286645	0.00048287
1363	TGGC	0.2286428	0.00048282
1364	CCAAG	0.2286328	0.0004828
1365	TTCGG	0.2286284	0.00048279
1366	63	0.2286226	0.00048278
1367	TGCGA	0.2285028	0.00048253
1368	ACTGC	0.2284344	0.00048238
1369	TACT	0.2284037	0.00048232
1370	91	0.2283482	0.0004822
1371	229	0.2282771	0.00048205
1372	TATTA	0.2280307	0.00048153
1373	CACC	0.2278166	0.00048108
1374	AAG	0.2277581	0.00048095
1375	CCC	0.2274322	0.00048026
1376	ATAA	0.2273573	0.00048011
1377	TAAA	0.2271962	0.00047977
1378	AT	0.2270377	0.00047943
1379	CGT	0.2267751	0.00047888
1380	GTGT	0.2259877	0.00047721
1381	GAGT	0.2259177	0.00047707
1382	120	0.2256633	0.00047653
1383	124	0.2256531	0.00047651
1384	65	0.2255568	0.0004763
1385	TTTTT	0.2253387	0.00047584
1386	TTTC	0.2251981	0.00047555
1387	AGCGG	0.2251917	0.00047553
1388	TATG	0.225175	0.0004755
1389	136	0.2251044	0.00047535
1390	56	0.2249725	0.00047507
1391	109	0.2248118	0.00047473
1392	CAT	0.2247936	0.00047469
1393	TCGAA	0.2247418	0.00047458
1394	51	0.2243905	0.00047384
1395	AGGTG	0.2243712	0.0004738
1396	CCAA	0.2240693	0.00047316
1397	CCTTG	0.2239734	0.00047296
1398	CTCAC	0.2236364	0.00047225
1399	144	0.223527	0.00047202
1400	GAATG	0.223309	0.00047156
1401	GTCGA	0.223207	0.00047134
1402	CGGGC	0.2231709	0.00047127
1403	TTCA	0.2228248	0.00047054
1404	AATGA	0.2227828	0.00047045
1405	134	0.222604	0.00047007
1406	GCGT	0.2224814	0.00046981

1407	228	0.2223091	0.00046945
1408	137	0.2222966	0.00046942
1409	129	0.2222492	0.00046932
1410	CAAG	0.221831	0.00046844
1411	59	0.221436	0.0004676
1412	57	0.2207987	0.00046626
1413	TTT	0.2206618	0.00046597
1414	69	0.2204485	0.00046552
1415	115	0.2200962	0.00046477
1416	TC	0.2200808	0.00046474
1417	ATA	0.219955	0.00046448
1418	CGGG	0.2198637	0.00046428
1419	GA	0.219687	0.00046391
1420	TGCGG	0.219591	0.00046371
1421	TCGG	0.2193562	0.00046321
1422	ACGCC	0.2191295	0.00046273
1423	CGAG	0.2191074	0.00046269
1424	21	0.2189945	0.00046245
1425	CGTTC	0.2181603	0.00046069
1426	chromosor	0.2180839	0.00046052
1427	TCC	0.2179838	0.00046031
1428	CCTTT	0.2179705	0.00046028
1429	34	0.2177246	0.00045977
1430	CGTT	0.2172301	0.00045872
1431	CTG	0.2170303	0.0004583
1432	GTCC	0.2170024	0.00045824
1433	22	0.2168802	0.00045798
1434	CGGGA	0.2167379	0.00045768
1435	ATAT	0.2166992	0.0004576
1436	CCTC	0.2163774	0.00045692
1437	TTG	0.216038	0.0004562
1438	116	0.2160176	0.00045616
1439	AAGCT	0.2158537	0.00045581
1440	ATTAA	0.2157556	0.00045561
1441	GCC	0.2155602	0.00045519
1442	CATT	0.2152087	0.00045445
1443	GGGC	0.2151164	0.00045426
1444	GAGA	0.2150908	0.0004542
1445	GTACG	0.2149428	0.00045389
1446	TAT	0.2144727	0.0004529
1447	CCCT	0.2142349	0.0004524
1448	AGCTC	0.214004	0.00045191
1449	GGT	0.2136695	0.0004512
1450	54	0.2136617	0.00045119
1451	224	0.2135934	0.00045104
1452	GTTG	0.2130486	0.00044989
1453	GATAA	0.213029	0.00044985

1454	179	0.2129456	0.00044967
1455	TTC	0.2128753	0.00044953
1456	CAC	0.2127789	0.00044932
1457	ATAAC	0.2127682	0.0004493
1458	CTTA	0.2127252	0.00044921
1459	ATTTC	0.212642	0.00044903
1460	40	0.2125828	0.00044891
1461	ATGA	0.212273	0.00044825
1462	CTCGG	0.2118061	0.00044727
1463	TAAAA	0.2116189	0.00044687
1464	AGCA	0.2112657	0.00044613
1465	ATACG	0.2111255	0.00044583
1466	105	0.2107271	0.00044499
1467	AG	0.2104082	0.00044432
1468	CCT	0.2103487	0.00044419
1469	CCCG	0.2100012	0.00044346
1470	GCAG	0.2094911	0.00044238
1471	60	0.2093086	0.00044199
1472	TCATG	0.2091123	0.00044158
1473	TCCA	0.2090388	0.00044142
1474	AGCGA	0.2088571	0.00044104
1475	GTCGC	0.2086879	0.00044068
1476	TGA	0.2086798	0.00044067
1477	GGA	0.2084844	0.00044025
1478	52	0.2081709	0.00043959
1479	GCAGG	0.2080138	0.00043926
1480	AATC	0.2078815	0.00043898
1481	61	0.2077403	0.00043868
1482	CGCT	0.2077326	0.00043867
1483	35	0.207549	0.00043828
1484	AA	0.2075165	0.00043821
1485	ACGGG	0.2075113	0.0004382
1486	CCGC	0.207139	0.00043741
1487	131	0.2070227	0.00043717
1488	GACG	0.2069266	0.00043696
1489	CC	0.2066825	0.00043645
1490	15	0.206601	0.00043628
1491	CTGTC	0.2064504	0.00043596
1492	TGT	0.2064121	0.00043588
1493	GCGA	0.2060416	0.00043509
1494	AATT	0.2059341	0.00043487
1495	32	0.2056976	0.00043437
1496	175	0.2055137	0.00043398
1497	ACCC	0.2054913	0.00043393
1498	GC	0.2052564	0.00043344
1499	113	0.2050242	0.00043295
1500	GCCTG	0.2049174	0.00043272

1501	ATC	0.2048207	0.00043252
1502	68	0.2046536	0.00043216
1503	11	0.204328	0.00043148
1504	GCCC	0.2042493	0.00043131
1505	CACCT	0.2037682	0.00043029
1506	TTTCG	0.2036742	0.0004301
1507	TGCG	0.2033192	0.00042935
1508	GCCA	0.2032804	0.00042926
1509	CT	0.2031107	0.00042891
1510	TATA	0.2021573	0.00042689
1511	CGTCG	0.2020825	0.00042673
1512	ACT	0.2018523	0.00042625
1513	CCTCG	0.2017482	0.00042603
1514	TAGG	0.2015672	0.00042565
1515	73	0.2014259	0.00042535
1516	GAAC	0.2013032	0.00042509
1517	AATA	0.2011382	0.00042474
1518	GTGCT	0.2010828	0.00042462
1519	ATG	0.2009817	0.00042441
1520	5	0.2004382	0.00042326
1521	TTTT	0.2002665	0.0004229
1522	GGCG	0.2002486	0.00042286
1523	GTCT	0.1995756	0.00042144
1524	TGACT	0.1988972	0.00042001
1525	chromosor	0.1980057	0.00041813
1526	ATTTT	0.1978133	0.00041772
1527	CGTG	0.1978	0.00041769
1528	TGACG	0.197576	0.00041722
1529	TTTGA	0.1967347	0.00041544
1530	130	0.1965947	0.00041515
1531	GGCGG	0.1962545	0.00041443
1532	AAATA	0.1960501	0.000414
1533	CGCTC	0.1960076	0.00041391
1534	transcript_	0.1959829	0.00041385
1535	77	0.1958568	0.00041359
1536	GGCT	0.195812	0.00041349
1537	CTGT	0.1955879	0.00041302
1538	CTAT	0.1954626	0.00041276
1539	TTGA	0.1951908	0.00041218
1540	ATCGT	0.1948572	0.00041148
1541	TA	0.1947661	0.00041128
1542	16	0.1940049	0.00040968
1543	80	0.193618	0.00040886
1544	TACC	0.193344	0.00040828
1545	TTCT	0.19316	0.00040789
1546	42	0.1927584	0.00040704
1547	1	0.1924233	0.00040634

1548	CTTC	0.1917927	0.00040501
1549	ATTT	0.1916266	0.00040465
1550	CTT	0.1916138	0.00040463
1551	GTCGG	0.1912763	0.00040391
1552	CCGGT	0.1902284	0.0004017
1553	ATTC	0.190226	0.0004017
1554	GTA	0.1898762	0.00040096
1555	CCTT	0.1896225	0.00040042
1556	TCGAC	0.188858	0.00039881
1557	chromosor	0.1888048	0.0003987
1558	AGAT	0.1885551	0.00039817
1559	GGTT	0.1876649	0.00039629
1560	154	0.1871446	0.00039519
1561	GAG	0.1859993	0.00039277
1562	TGG	0.184874	0.0003904
1563	ATT	0.184312	0.00038921
1564	ATTG	0.1831341	0.00038672
1565	95	0.1827576	0.00038593
1566	CACA	0.1808509	0.0003819
1567	CAAAA	0.1793942	0.00037882
1568	AGTT	0.1790731	0.00037815
1569	ATGT	0.1789292	0.00037784
1570	CGAT	0.1771562	0.0003741
1571	GAT	0.1756764	0.00037097
1572	TT	0.1740005	0.00036743
1573	ACA	0.1739528	0.00036733
1574	23	0.1720943	0.00036341
1575	142	0.1709916	0.00036108
1576	GAGAG	0.1674907	0.00035369
1577	117	0.1647277	0.00034785
1578	149	0.1638289	0.00034595
1579	TGACA	0.1624984	0.00034315
1580	TAA	0.1596418	0.00033711
1581	AGA	0.1596212	0.00033707
1582	CCTG	0.155129	0.00032758

Table S3. DeeplncRNA Feature Importance.

Every feature is ranked according to its feature importance in the DeepLncRNA model. For each feature we show its rank and name, a numbered name represents an index referencing a motif in the CISBP-RNA binding protein database. We also show summary statistics for the three feature sets utilized which are the genomic, Kmer and RBP (RNA binding protein motif) features.

LncRNA	Ensembl ID	Localized	Reference
lincRNA-p21	ENSMUSG00000085912	Cytosol	(Barsotti & Prives 2010)
LINCMD1	ENSG00000225613	Cytosol	(Cesana et al. 2011)
NORAD	ENSG00000260032	Cytosol	(Tichon et al. 2016)
H19	ENSG00000130600	Cytosol	(Giovarelli et al. 2014)
NKILA	ENSG00000278709	Cytosol	(Liu et al. 2015)
U50HG	ENSG00000203875	Cytosol	(Derrien et al. 2012)
DANCR	ENSG00000226950	Cytosol	(Lennox & Behlke 2016)
OIP5-AS1	ENSG00000247556	Cytosol	(Lennox & Behlke 2016)
SNHG1	ENSG00000255717	Cytosol	(Carlevaro-Fita et al. 2016)
BORG		Nuclear	(Zhang et al. 2014)
XIST	ENSG00000229807	Nuclear	(Cohen & Panning 2007)
Malat1	ENSG00000251562	Nuclear	(Cabili et al. 2015)
NEAT1	ENSG00000245532	Nuclear	(Jiang et al. 2017)
MEG3	ENSG00000214548	Nuclear	(Mondal et al. 2015)
DLX6-AS1	ENSG00000231764	Nuclear	(Feng et al. 2006)
GAS5	ENSG00000234741	Nuclear	(Derrien et al. 2012)
PINCR	ENSG00000224294	Nuclear	(Chaudhary et al. 2017)
UCHL1-AS1	ENSG00000251173	Nuclear	(Carrieri et al. 2012)
TUG1	ENSG00000253352	Dual	(Lennox & Behlke 2016)
CasC7	ENSG00000123908	Dual	(Lennox & Behlke 2016)
HOTAIR	ENSG00000228630	Dual	(Lennox & Behlke 2016)

Table S4. Manually Curated lncRNAs with experimentally verified subcellular localizations.

For each selected lncRNA we show the lncRNAs gene name, Ensembl ID, subcellular localization and the study in which it was experimentally determined. Dual localization indicates the lncRNA was present in both subcellular fractions.

References

Barsotti, A.M. & Prives, C., 2010. Noncoding RNAs: The missing “linc” in p53-mediated repression. *Cell*, 142(3), pp.358–360.

Cabili, M.N. et al., 2015. Localization and abundance analysis of human lncRNAs at single-cell and single-molecule resolution. *Genome Biology*, 16(20), pp.1–16.

Carlevaro-Fita, J. et al., 2016. Cytoplasmic long noncoding RNAs are frequently bound to and degraded at ribosomes in human cells. *Rna*, pp.1–16.

Carrieri, C. et al., 2012. Long non-coding antisense RNA controls Uchl1 translation through an

embedded SINEB2 repeat. *Nature*, 491(7424), pp.454–457.

Cesana, M. et al., 2011. A long noncoding RNA controls muscle differentiation by functioning as a competing endogenous RNA. *Cell*, 147(2), pp.358–369.

Chaudhary, R. et al., 2017. Prosurvival long noncoding RNA PINCR regulates a subset of p53 targets in human colorectal cancer cells by binding to Matrin 3. *eLife*, 6, pp.1–32.

Cohen, H.R. & Panning, B., 2007. XIST RNA exhibits nuclear retention and exhibits reduced association with the export factor TAP/NXF1. *Chromosoma*, 116(4), pp.373–383.

Derrien, T. et al., 2012. The GENCODE v7 catalog of human long noncoding RNAs: Analysis of their gene structure, evolution, and expression. *Nature*, 22(9), pp.1775–1789.

Feng, J. et al., 2006. The Evf-2 noncoding RNA is transcribed from the Dlx-5/6 ultraconserved region and functions as a Dlx-2 transcriptional coactivator. *Genes and Development*, 20(11), pp.1470–1484.

Giovarelli, M. et al., 2014. H19 long noncoding RNA controls the mRNA decay promoting function of KSRP. *Proceedings of the National Academy of Sciences*, 111(47), pp.E5023–E5028.

Jiang, L. et al., 2017. NEAT1 scaffolds RNA-binding proteins and the Microprocessor to globally enhance pri-miRNA processing. *Nature Structural and Molecular Biology*, 24(10), pp.816–824.

Lennox, K.A. & Behlke, M.A., 2016. Cellular localization of long non-coding RNAs affects silencing by RNAi more than by antisense oligonucleotides. *Nucleic Acids Research*, 44(2), pp.863–877.

Liu, B. et al., 2015. A Cytoplasmic NF-κB Interacting Long Noncoding RNA Blocks IκB Phosphorylation and Suppresses Breast Cancer Metastasis. *Cancer Cell*, 27(3), pp.370–381.

Mondal, T. et al., 2015. MEG3 long noncoding RNA regulates the TGF-β pathway genes through formation of RNA-DNA triplex structures. *Nature Communications*, 6.

Tichon, A. et al., 2016. A conserved abundant cytoplasmic long noncoding RNA modulates repression by Pumilio proteins in human cells. *Nature Communications*, 7, pp.1–10.

Zhang, B. et al., 2014. A novel RNA motif mediates the strict nuclear localization of a long noncoding RNA. *Molecular and cellular biology*, 34(12), pp.2318–2329.
